# NSD2 E1099K drives relapse in pediatric acute lymphoblastic leukemia by disrupting 3D chromatin organization

**DOI:** 10.1186/s13059-023-02905-0

**Published:** 2023-04-04

**Authors:** Sonali Narang, Nikki A. Evensen, Jason Saliba, Joanna Pierro, Mignon L. Loh, Patrick A. Brown, Pandurang Kolekar, Heather Mulder, Ying Shao, John Easton, Xiaotu Ma, Aristotelis Tsirigos, William L. Carroll

**Affiliations:** 1 Perlmutter Cancer Center, NYU Langone Health, Smilow 1211, 560 First Avenue, New York, NY 10016 USA; 2 Northwell Health, Staten Island University Hospital, Staten Island, NY USA; 3 Department of Pediatrics, Benioff Children’s Hospital and The Helen Diller Family Comprehensive Cancer Center University of California, San Francisco, San Francisco, CA USA; 4 The Sidney Kimmel Comprehensive Cancer Center at Johns Hopkins, Johns Hopkins University School of Medicine, Baltimore, MD USA; 5 Department of Computational Biology, St. Jude Children’s Research Hospital, Memphis, TN USA; 6 Department of Pathology, NYU Langone Health, New York, NY USA; 7 Perlmutter Cancer Center, NYU Langone Health, Science Building 800, 435 East 30th Street, New York, NY 10016 USA; 8 Department of Pediatrics, NYU Langone Health, New York, NY USA; 9 Division of Pediatric Hematology/Oncology, NYU Langone Health, New York, NY USA

**Keywords:** Relapsed acute lymphoblastic leukemia, NSD2, Clonal evolution, Chromatin architecture

## Abstract

**Background:**

The NSD2 p.E1099K (EK) mutation is shown to be enriched in patients with relapsed acute lymphoblastic leukemia (ALL), indicating a role in clonal evolution and drug resistance.

**Results:**

To uncover 3D chromatin architecture-related mechanisms underlying drug resistance, we perform Hi-C on three B-ALL cell lines heterozygous for NSD2 EK. The NSD2 mutation leads to widespread remodeling of the 3D genome, most dramatically in terms of compartment changes with a strong bias towards A compartment shifts. Systematic integration of the Hi-C data with previously published ATAC-seq, RNA-seq, and ChIP-seq data show an expansion in H3K36me2 and a shrinkage in H3K27me3 within A compartments as well as increased gene expression and chromatin accessibility. These results suggest that NSD2 EK plays a prominent role in chromatin decompaction through enrichment of H3K36me2. In contrast, we identify few changes in intra-topologically associating domain activity. While compartment changes vary across cell lines, a common core of decompacting loci are shared, driving the expression of genes/pathways previously implicated in drug resistance. We further perform RNA sequencing on a cohort of matched diagnosis/relapse ALL patients harboring the relapse-specific NSD2 EK mutation. Changes in patient gene expression upon relapse significantly correlate with core compartment changes, further implicating the role of NSD2 EK in genome decompaction.

**Conclusions:**

In spite of cell-context-dependent changes mediated by EK, there appears to be a shared transcriptional program dependent on compartment shifts which could explain phenotypic differences across EK cell lines. This core program is an attractive target for therapeutic intervention.

**Supplementary Information:**

The online version contains supplementary material available at 10.1186/s13059-023-02905-0.

## Background

While the overall outcome with children with acute lymphoblastic leukemia (ALL) has improved dramatically, up to 20% of patients relapse making ALL one of the leading causes of cancer-related death in children [1, 2]. Genome-wide profiling studies have shown enrichment of mutations at relapse including somatic alterations in epigenetic regulators such as *MSH6*, *SETD2*, and *NSD2* [3]. *NSD2* is a key histone methyltransferase involved in monomethylation and dimethylation of lysine 36 of histone 3 (H3K36), a mark associated with active transcription [4]. Specifically, the recurrent gain of function mutation p.E1099K (EK) has been found to be enriched in patients with relapsed ALL [5].

The NSD2 EK mutation results in increased methyltransferase activity leading to a global increase in H3K36me2 levels (active mark) as well as the concomitant inhibition of EZH2-mediated H3K27me3 levels (repressive mark) in pediatric ALL [6, 7]. Our previous studies have shown knockdown of NSD2 in EK mutated B-ALL cell lines resulted in decreased proliferation, decreased clonal growth, and increased sensitivity to cytotoxic chemotherapeutic agents with no effect on NSD2 wildtype lines [5]. Other work also demonstrated changes in growth and clonogenicity as well as cellular adhesion upon CRISPR-mediated reversion of EK to wildtype in NSD2 mutated lines [7]. Interestingly, RNA-seq data from both studies revealed variable transcriptional reprogramming upon loss of NSD2 in different cell line models. While some common pathways were identified, such as cell adhesion and Rap1 signaling, collectively the data suggests cell-context-specific changes occur in response to mutated NSD2. Importantly, we also demonstrated minimal overlap in chromatin accessibility changes upon NSD2 knockdown in the three EK harboring cell lines [5]. Our current work addresses the transcriptional and chromatin accessibility heterogeneity observed as a result of the NSD2 EK mutation.

In this study, we investigate the role 3D genome organization plays in EK-mediated relapse. 3D genome organization refers to the strategic positioning of regulatory elements to regions best suited for the regulation of genome function. The organizational hierarchy is made up of multiscale structural units such as chromosomal territories, A/B compartments, topologically associating domains (TADs), and chromatin loops each of which play an important role in regulating gene expression [8, 9]. At the Mb scale, chromosomes are spatially divided into two major domains, A and B compartments, that correspond to active and inactive chromatins, respectively [10, 11]. At the sub-Mb scale, the genome can be further subdivided into highly self-interacting chromatin units referred to as TADs [10, 12, 13]. TADs play a major role in the regulation of gene expression by restricting the influence of regulatory elements to genes within the same TAD as well as insulating them from interactions with neighboring domains [14].

Dysregulation of higher-order genomic architecture in several disease models has been linked to changes in the epigenetic landscape [15–18]. In multiple myeloma, expansion of H3K26me2 and shrinkage of H3K27me3 domains as a result of NSD2 overexpression were shown to be linked to chromatin changes in TADs and CTCF loops as well as disruptions in gene expression [15]. In lymphoma, profound decompaction of the genome as a result of disruption in H1 function was shown to drive changes in the epigenetic landscape including the gain of H3K36me2 and loss of H3K27me3 leading to the aberrant expression of normally silenced stem cell-associated genes [16]. In B-ALL, however, NSD2-mediated context-dependent phenotypic changes have yet to be linked to the dysregulation of higher-order structures.

To uncover 3D chromatin architecture-related mechanisms underlying drug resistance, we performed Hi-C on three B-ALL cell lines heterozygous for NSD2 EK (RS4;11, RCH-ACV, SEM) and assessed changes between NSD2 Low (knockdown) and NSD2 High (mutant) cell lines. NSD2 EK led to the widespread remodeling of the 3D genome, most dramatically in terms of A/B compartment changes with a strong bias towards A compartment shifts. Systematic integration of the Hi-C data with previously published ATAC-seq, RNA-seq and ChIP-seq data showed an expansion in H3K36me2 and a shrinkage in H3K27me3 marks within A compartments as well as increased gene expression and chromatin accessibility. These results suggest that NSD2 EK plays a prominent role in chromatin decompaction through enrichment of H3K36me2 epigenetic marks. In contrast, we identified few changes in intra-TAD activity suggesting that EK-mediated transcriptional changes occur through a remarkable dependence on compartmentalization. While compartment changes varied across cell lines, a common core of decompacting loci were shared driving the expression of genes/pathways previously implicated in drug resistance. To validate these findings beyond in vitro models, we performed RNA-seq on a cohort of matched diagnosis/relapse ALL patients harboring the relapse-specific NSD2 EK mutation. Patient gene expression changes upon relapse significantly correlated with core compartment changes further implicating NSD2 EK in genome decompaction. In spite of cell-context-dependent changes mediated by EK, there appears to be a shared transcriptional program dependent on compartment shifts which could explain phenotypic differences across EK cell lines. This core program is an attractive target for therapeutic intervention.

## Results

### NSD2 drives widespread changes in A/B compartments

**Fig. 1:**
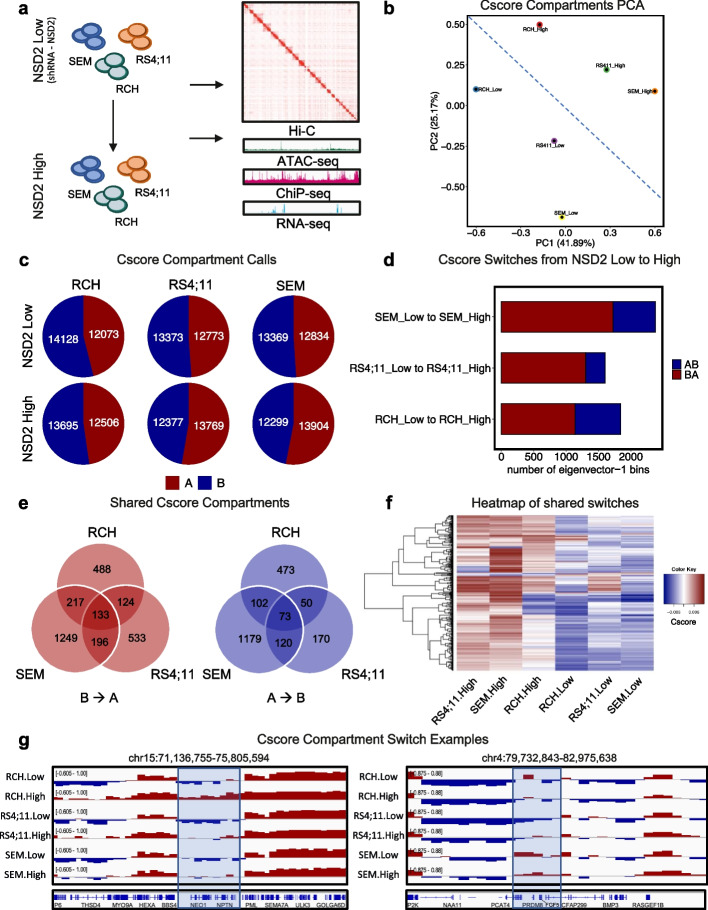
NSD2 EK drives A/B compartment reorganization. **a** Schematic demonstrating overall study design. **b** PCA of A/B compartment calls with Cscore. **c** Pie chart showing numbers of A and B compartment calls for each cell line in NSD2 Low and High cell lines. **d** Bar plot showing number of compartment switches for each cell line from NSD2 Low to NSD2 High. **e** Venn diagram showing overlap of switching compartments between three B-ALL cell lines, B to A and A to B switches (left and right respectively). **f** Heatmap representation of the cscore (first eigenvector (PC1) of the correlation matrix) for genomic bins undergoing compartment switches in at least 2 of the 3 cell lines due to NSD2 EK. **g** IGV tracks of an example of B to A compartment switch shared by all three cell lines at the *NEO1* locus (left). IGV tracks of an example of a B to A compartment switch specific to RS4;11 cell line at the *PRDM8* and *FGF5* locus (right)

We performed Hi-C and systematically integrated it with previously published and new ATAC-seq, RNA-seq, and ChIP-seq data from three B-ALL cell lines heterozygous for NSD2 EK (RS4;11, RCH-ACV, SEM) either expressing a NSD2 targeting shRNA or a non-targeting shRNA henceforth referred to as NSD2 Low and High cell lines [5] (Fig 1a). In this study, we assessed changes in these three cell lines from NSD2 Low (knockdown) to NSD2 High (mutant) as NSD2 EK is a relapse-enriched mutation in patients and results in the overactive form of NSD2. Hi-C was performed using the Arima Kit, and Hi-C data was processed by our Hi-C-bench platform and showed alignment rates with a high number of usable intra-chromosomal long-range read-pairs (~100 million) [19] (Additional file 1: Fig. S1a). We first assessed compartmentalization with the CscoreTool algorithm by calling A/B compartments [20]. Principal component analysis (PCA) of compartment Cscores revealed a clear separation between NSD2 Low and High cell lines (Fig. 1b). Cscore compartment calls showed similar percentages of A/B compartments in NSD2 Low and NSD2 High cell lines (Fig. 1c).

We next examined changes in A/B compartments between NSD2 Low and NSD2 High cell lines. We observed ~7.39% compartment switching across the three cell lines (Fig. 1d). Overall, a greater number of compartment switches occurred from B to A with 4160 regions switching from B to A and 1661 regions switching from A to B. Of the switching compartments, we identified 219 shared switches across the three cell lines from B to A and 50 from A to B (Fig. 1e). 22.40% (932/4,160) of B to A switches and 16.20% (269/1,661) A to B switches were shared by at least 2 cell lines (Fig. 1f). One such compartment switch from B to A shared by all three cell lines involved the Neogenin 1 (*NEO1*) gene locus, which has been implicated in cell adhesion (Fig. 1g left panel) [21]. Several other notable genes shared by at least two lines have been previously implicated in cancer, including Epidermal Growth Factor Receptor Kinase Substrate 8 (*EPS8)* which has been associated with poor prognosis in ALL and is known to regulate proliferation and apoptosis [22], *FAM92A* (also known as *BARMR1*) which has been negatively correlated with prognosis in AML and shown to promote proliferation and colony formation [23], and insulin receptor substrate 1 (*IRS1)* which can activate PI3K/AKT/mTOR, B-catenin, and MAPK has been a target for inhibiting MAPK in ALL [24, 25]. Additionally, we identified cell-line-specific switches including a B to A switch at the PR Domain Zinc Finger Protein 8 (*PRDM8*) and *FGF5* gene locus in RS4;11 (Fig. 1g right panel) as well as guanosine nucleotide-binding protein Q gene (*GNAQ*) in RCH.

### NSD2-related compartment switches alter gene expression in a cell-context-dependent manner

**Fig. 2:**
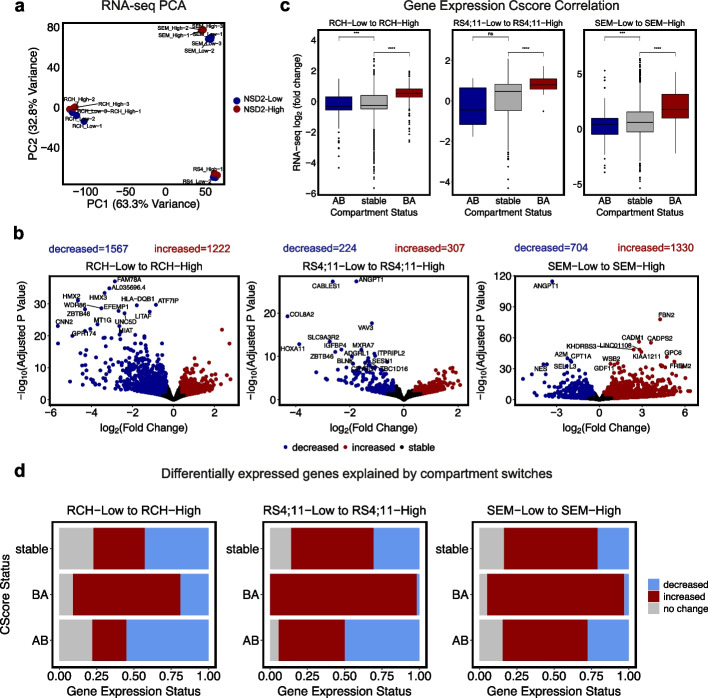
NSD2 EK-related B to A compartment switches correlate with gene expression changes. **a** PCA representation of triplicate RNA-seq data showing top 1000 genes. **b** Volcano plots demonstrating differentially expressed genes (abs(L2FC) > 0.32, *p*-value < 0.05) per cell line from NSD2 Low to NSD2 High with top 10 labeled. **c** Correlation boxplots of gene expression changes and compartment switch status (AB, BA, stable) for each cell line. **d** Association barplots showing fraction of genes (increased, decreased, stable) at compartment switches (AB, BA, stable)

To understand how NSD2 expression alters gene expression, we first analyzed previously published RNA-seq data from NSD2 Low and NSD2 High cell lines [5]. PCA revealed that NSD2 Low and NSD2 High replicates separated into distinct cell-type specific clusters demonstrating transcriptional heterogeneity (Fig. 2a). As previously described, we observed that NSD2 EK leads to the deregulation of several genes across all three cell lines (Fig. 2b). NSD2 was notably confirmed to be upregulated among other genes (Fig. 2b). Overlap analysis also revealed changes in gene expression to be predominantly cell-context dependent with minimal overlap between cell lines (Additional file 1: Fig. S2a). Only 1.24% of differentially expressed genes were shared between the three cell lines.

To examine the relationship between alterations in A/B compartments and gene expression, compartment switches were categorized as A to B, B to A, or stable and then assessed for changes in gene expression. BA compartment switches significantly correlated with upregulated genes. Notably, AB compartment switches did not correlate with downregulated genes to a similar extent (Fig. 2c). Likewise, we observed that the B to A compartment switches were characterized by a significant fraction of upregulated genes (>75% in RS4;11 and SEM) whereas A to B compartment switches did not show as strong of a relationship with downregulated genes (Fig. 2d). This data suggests that NSD2 EK-mediated reorganization of compartments from B to A results in gene expression changes.

**Fig. 3:**
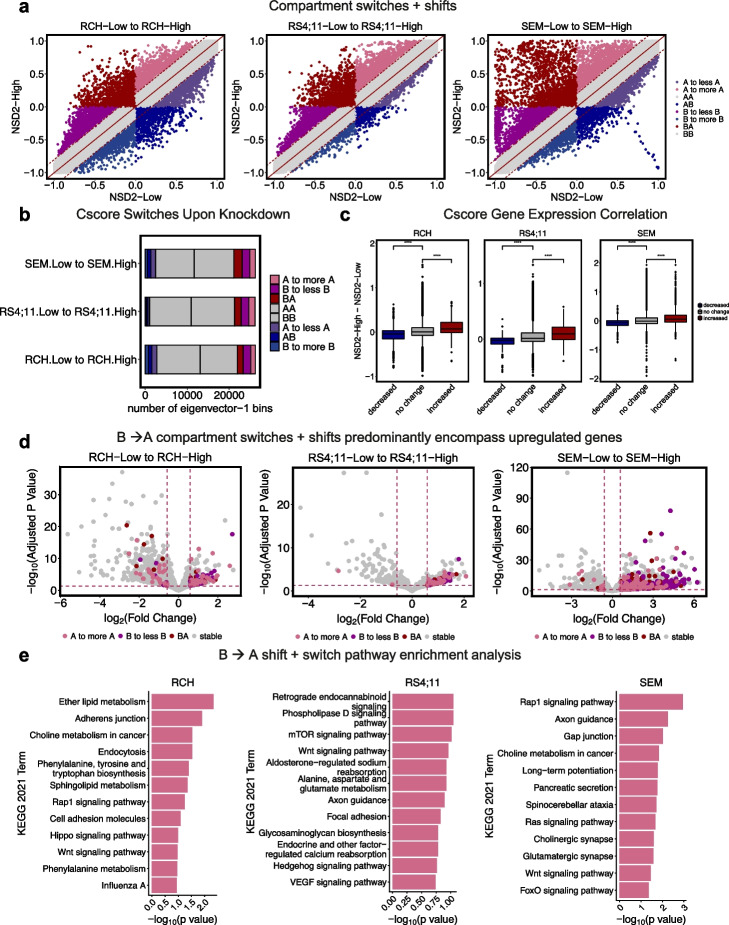
NSD2 EK-related B to A compartment switches and shifts correlate with gene expression changes. **a** Scatter plot demonstrating strategy for calling 6 categories of compartment switches and shifts (A to B, and B to A compartment switches; A to less A, A to more A, B to less B, and B to more B compartment shifts). **b** Bar plot showing number of compartment switches and shifts for each cell line from NSD2 Low to NSD2 High. **c** Correlation boxplots of compartment cscore and gene expression changes (abs(L2FC) > 0.32, *p*-value 0.05) for each cell line. **d** Volcano plots demonstrating differentially expressed genes (abs(L2FC) > 0.32, *p*-value < 0.05) highlighted by compartment switch or shift (A to less A, A to B, B to more B, or stable). **e** KEGG 2021 pathway enrichment analysis of genes differentially expressed (L2FC < −0.32, *p*-value < 0.05) at B to A compartment switches or A to more A and B to less B compartment shifts per cell line

To expand our analysis, we incorporated compartment shifts in addition to compartment switches (Fig. 3a). Shifts include A to more A, B to more B, A to less A, and B to more B. The addition of compartment shifts revealed 26.06% compartment changes between NSD2 Low and NSD2 High cell lines (Fig. 3b). Further exploring concordance with gene expression revealed that compartment changes significantly correlated with gene expression changes (Fig. 3c). We also identified that the majority of B to A switches and shifts were made up of significantly upregulated genes (Fig. 3d; L2FC < 0.32, *p*-value < 0.05), whereas A to B switches and shifts did not demonstrate a clear pattern similar to our findings with switches alone (Fig. 3d, Additional file 1: Fig. S2b).

To further explore this concordance between compartment and gene expression changes, we assessed the number of differentially expressed genes explained by compartment changes. Approximately 7.60% of genes upregulated could be explained by compartments that switched from B to A (Additional file 1: Fig. S3). With the addition of compartment shifts, 20.00% of upregulated genes could be explained by compartment switches and shifts (Additional file 1: Fig. S3). In contrast, only 1.20% of downregulated genes could be explained by compartment changes. Ascribing a concordance score to each compartment shift and switch revealed that concordance was observed specifically in those compartments that decompacted (Additional file 1: Fig. S2c).

Lastly, to better understand the impact these changes have on downstream signaling, we performed pathway enrichment analysis for those differentially expressed genes that were associated with either a compartment switch or shift per cell line (Fig. 3e). Interestingly, cancer-related pathways previously identified by RNA-seq or ChIP-seq analysis [5, 26] were also found in our compartment-based analysis including Rap1, Ras, Phosphatidylinositol 3-kinase, and calcium signaling. Furthermore, these pathways were shared by at least two of the three cell lines providing evidence that the existence of a core of decompacting loci can explain previously described shared phenotypes such as proliferation [5].

Additionally, we performed a subcompartments analysis by applying SNIPER to our Hi-C datasets as a complementary analysis to the compartments switch and shift analysis (Additional file 1: Fig. S4 and S5). SNIPER (Subcompartment iNference using Imputed Probabilistic ExpRessions) is a computational method that is based on denoising autoencoder and multilayer perceptron classifier to infer subcompartments using typical Hi-C datasets with moderate coverage. SNIPER reveals subcompartments using moderate coverage Hi-C datasets [27].

We first present the number of subcompartments classified as A1, A2, B1, B2, and B3 with SNIPER for each of our cell lines at NSD2 Low and NSD2 High (Additional file 1: Fig. S4a). Next, we did a head-to-head comparison of the SNIPER subcompartments analysis with our initial analysis using Cscore. For this analysis, we identified the fraction of Cscore A or B compartments that overlapped the SNIPER subcompartments (Additional file 1: Fig. S4b). This data revealed that the B1 subcompartment was made up of mostly Cscore compartment A suggesting a discrepancy between the two methods. Moving forward, we excluded subcompartment B1 in order to investigate the SNIPER subcompartment analysis further. We present the number of subcompartments classified as A1, A2, B2, and B3 with SNIPER for each of our cell lines at NSD2 Low and NSD2 High (Additional file 1: Fig. S4c).

We next examined changes in A/B subcompartments between NSD2 Low and NSD2 High cell lines (Additional file 1: Fig. S4d). We noticed that both the RS4;11 and SEM cell lines showed a greater number of B to A subcompartment switches than A to B from NSD2 Low to NSD2 High which was consistent with our Cscore analysis. Next, we further classified subcompartment calls into shifts in addition to subcompartment switches. Activating subcompartment shifts include B3 to B1, B3 to B2, B2 to B1, and A2 to more A1. Deactivating subcompartment shifts include B1 to B3, B2 to B3, B1 to B2, and A1 to more A2. With this data, we identified the fraction of SNIPER subcompartment switches and shifts that overlapped the Cscore compartments switches and shifts (Additional file 1: Fig. S4e). Overall, this data showed similar trends in Cscore compartment and SNIPER subcompartment switches and shifts.

Lastly, to examine the relationship between alterations in SNIPER subcompartments and gene expression, subcompartment switches were categorized as A to B, B to A, or stable and then assessed for changes in gene expression. B to A subcompartment switches seemed to more significantly correlate with upregulated genes than A to B subcompartment switches did with downregulated genes as we saw with the Cscore and gene expression analysis (Additional file 1: Fig. S5a). To further examine the relationship between alterations in SNIPER subcompartments and gene expression, we added subcompartment shifts in addition to switches. We identified that the majority of B to A switches and shifts were made up of significantly upregulated genes (Additional file 1: Fig. S5b (bottom row)), whereas A to B switches and shifts did not demonstrate a clear pattern similar to our findings with Cscore switches and shifts (Additional file 1: Fig. S5b (top row)).

### NSD2 EK-related compartment switches associate with chromatin accessibility changes

**Fig. 4:**
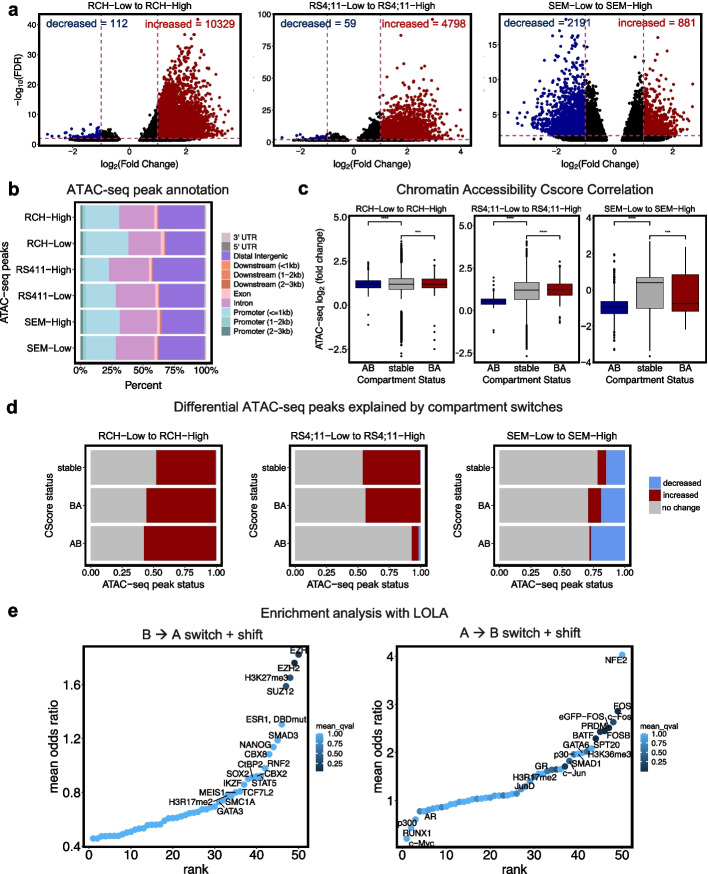
NSD2 knockdown leads to cell-type specific alterations in chromatin accessibility. **a** Volcano plots demonstrating differentially accessible regions from NSD2 Low to NSD2 High per cell line (abs(L2FC) > 1, FDR < .01). **b** Bar plot showing distribution of annotated ATAC-seq peaks for each NSD2-Low and NSD2-High cell line. **c** Correlation boxplots of chromatin accessibility changes and compartment switches (AB, BA, stable) for each cell line. **d** Association barplots showing fraction of ATAC-seq peaks (increased, decreased, stable) at compartment switches (AB, BA, stable). **e** Enrichment analysis using LOLA for increased ATAC-seq peaks concordant with increased gene expression and B to A compartment switches and shifts (left) and decreased ATAC-seq peaks concordant with decreased gene expression and A to B compartment switches and shifts (right)

To explore how NSD2 EK alters chromatin accessibility, ATAC-seq was performed on the NSD2 Low and NSD2 High cell lines. As previously described [5], NSD2 leads to the restructuring of a modest number of peaks across all three cell lines. This data showed a significant gain of ATAC-seq peaks for two of the three cell lines whereas SEM showed a paradoxical bias towards decreased accessibility (Fig. 4a). PCA and heatmap revealed that NSD2 Low and NSD2 High replicates separated into distinct cell-type-specific clusters demonstrating chromatin accessibility heterogeneity (Additional file 1: Fig. S6a,b). Overlap analysis also revealed changes to be predominantly cell-context dependent with minimal overlap between cell lines (Additional file 1: Fig. S6c). Additionally, we observed a shift in the distribution of the ATAC-seq peaks from promoter regions towards intergenic regions as has been previously shown with H3K36me2 peak distribution (Fig. 4b) [7, 28].

To examine the relationship between alterations in A/B compartments and chromatin accessibility, compartment switches were categorized as A to B, B to A, or stable and then assessed for changes in accessibility. Chromatin accessibility data did not show strong correlation with compartment data across the three cell lines. The majority of differential peaks were increased regardless of directionality of compartment changes for RCH-ACV and RS4;11 lines. The opposite was true for SEM, which showed decreased accessibility overall (Fig. 4c). Overall, SEM demonstrates a very different chromatin state than RCH-ACV and RS4;11 cell lines. Compartment switches were characterized by predominantly stable ATAC-seq peaks. We also noticed significant variability in the association with differential peaks across the three cell lines (Fig. 4d).

Exploring this link further, we identified concordant changes in A/B compartments, gene expression, and chromatin accessibility at the *NEO1* and *PRDM8/FGF5* loci. Along with the shared compartment switch from B to A in all three cell lines at the *NEO1* locus, we also identified concordant increases in chromatin accessibility and gene expression shown by genome browser tracks (Additional file 1: Fig. S6d). Similarly, along with the RS4;11 cell-type specific compartment switch from B to A at the *PRDM8/FGF5* locus, we identified concordant increases in chromatin accessibility and gene expression in RS4;11 cells shown by genome browser tracks (Additional file 1: Fig. S6e).

To identify key regulators of transcription attributed to compartment switches, we performed an enrichment analysis with LOLA using genomic loci with concordant increased ATAC-seq peaks, increased gene expression, and B to A compartment switches and shifts, and genomic loci with concordant decreased ATAC-seq peaks, decreased gene expression, and A to B compartment switches and shifts (Fig. 4e). EZH2 and H3K27me3, involved in repression, were among the top hits for sites with B to A compartment changes. Notably, factors linked to stem cell functionality, including NANOG, SUZ12, SOX2, and MEIS1, were also enriched [29].

### NSD2 EK leads to few intra-TAD activity changes

**Fig. 5:**
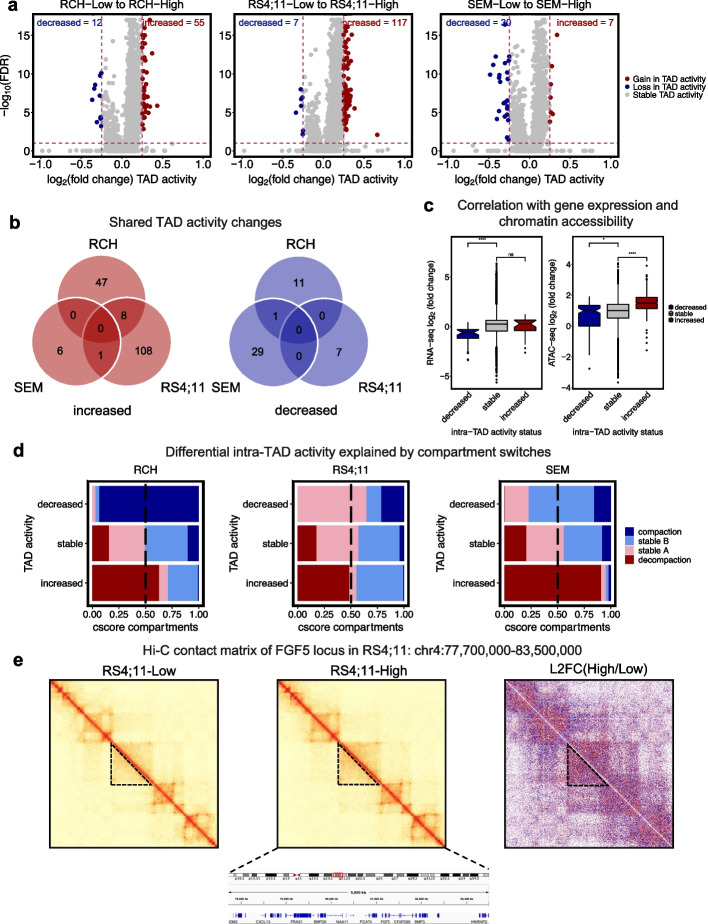
NSD2 knockdown has minimal impact on intra-TAD activity. **a** Volcano plots demonstrating differential intra-TAD activity from NSD2 Low to NSD2 High per cell line (abs(L2FC) > 0.25, FDR < .01). **b** Venn diagram showing overlap of differential TADs between three B-ALL cell lines, decreased and increased (left and right respectively). **c** Correlation boxplots showing gene expression and chromatin accessibility changes within differential TADs. **d** Association barplots showing fraction of compartment switches and shifts (compaction, stable B, stable A, and decompaction) at intra-TAD activity changes (increased, decreased, or stable). **e** Example of a TAD activity alteration using Hi-C contact matrices for RS4;11 at the *FGF5* locus showing an increase in intra-TAD activity (NSD2-Low, NSD2-High, and L2FC(High/Low) from left to right)

Following our compartment analysis, we investigated TAD activity changes between NSD2 Low and NSD2 High cell lines. Assessing mean TAD activity across samples revealed that the bulk of TADs remain stable across the three cell lines (Additional file 1: Fig. S7a). Comparison of intra-TAD activity between NSD2 Low and NSD2 High cell lines identified several statistically significant increases and very few decreases across all three cell types (Fig. 5a, Additional file 1: Fig. S7b; FDR < 0.1 and abs(L2FC) > 0.25). Of the differential TADs, we identified no shared changes in TAD activity between the three cell lines suggesting that changes in TAD activity are cell-context dependent (Fig. 5b).

To investigate whether changes in intra-TAD activity associated with changes in gene expression and chromatin accessibility, we performed differential expression analysis (abs(L2FC) >0.32, *p* <0.05) and differential chromatin accessibility analysis (abs(L2FC) >0.32, FDR <0.01) within differentially active TADs. Although few differential TADs were identified, integration of gene expression changes and chromatin accessibility changes with differentially active TADs indicated a significant correlation in both cases (Fig. 5c). In addition, we observed that increases in TAD activity predominantly occurred in regions with decompacting compartment switches and shifts and decreases in TAD activity predominantly occurred in regions with compacting compartment switches and shifts (Fig. 5d). In addition to earlier data, we identified a cell-type-specific increase in TAD activity at the *FGF5* locus in RS4;11 concordant with compartment switch, chromatin accessibility, and gene expression as shown by the contact matrix at NSD2 Low, NSD2 High, and L2FC contact matrix (High/Low) (Fig. 5e).

### NSD2 mutant patient samples reflect B-ALL cell line data

**Fig. 6:**
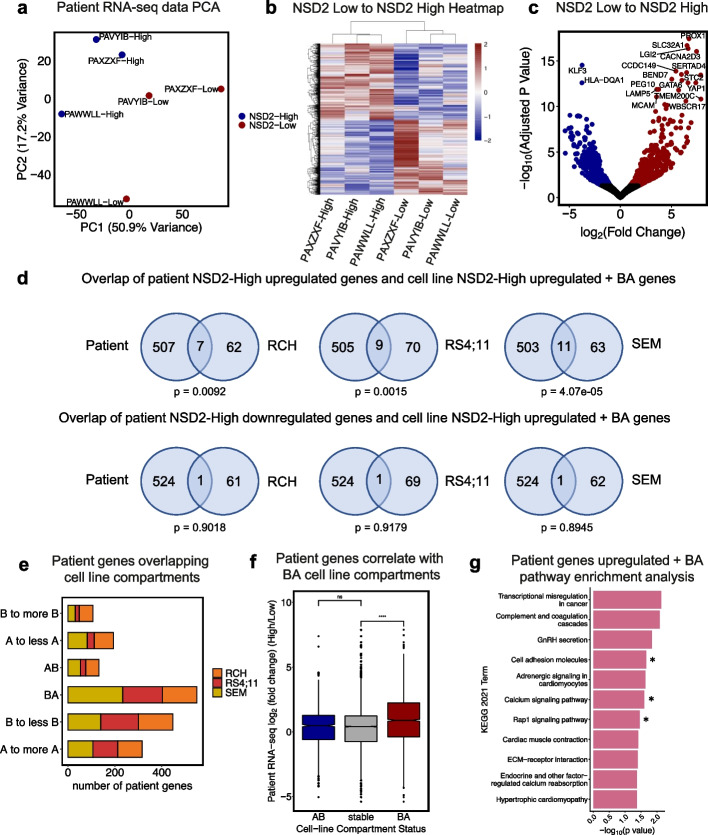
NSD2 mutant patient samples reflect B-ALL cell line data. **a** PCA of 3 matched diagnosis (NSD2-Low) and relapse (NSD2-High) patient pair gene expression data. **b** Heatmap of significantly expressed genes upon relapse (from NSD2 Low to NSD2 High: abs(L2FC) > 0.32, *p*-adjusted < 0.05). **c** Volcano plot demonstrating differentially upregulated genes highlighted in red and downregulated genes in blue(abs(L2FC) > 0.32, *p*-adjusted < 0.05). **d** Venn diagrams demonstrating significant overlap of patient NSD2-High upregulated genes and cell line NSD2-High upregulated genes with BA compartment switches and insignificant overlap of patient NSD2-High downregulated genes and cell line NSD2-High upregulated genes with BA compartment switches (one-tailed Fisher’s exact test). **e** Barplot demonstrating number of patient genes overlapping cell line compartment switches and shifts colored by cell line. **f** Correlation boxplot showing patient gene expression changes within cell line compartment changes (NSD2-High/NSD2-Low). **g** KEGG 2021 pathway enrichment analysis of patient genes upregulated in NSD2-High patients (abs(L2FC) > 0.32, *p*-adjusted < 0.05) that overlap with cell line B to A compartment switches, B to less B, and A to more A compartment shifts from any of the three cell lines. Starred pathways represent pathways previously shown in NSD2 models

To examine how relapse-enriched NSD2 EK mutation behaves in patient samples, we acquired expression data from three matched diagnosis (NSD2 Low) and relapse (NSD2 High) patient pairs (Additional file 1: Fig. S8). PCA revealed that NSD2 High samples separated from the NSD2 Low samples by the first principal component (Fig. 6a). Heatmap and volcano plots both demonstrate significant NSD2-mediated changes in gene expression with 525 increased and 514 decreased genes (Fig. 6b,c; abs(L2FC) >0.32, *p*-adj <0.05). To test our hypothesis that EK-mediated reorganization of compartments affects gene expression in relapse, we compared gene expression data from the patient samples to expression and compartment data from the three cell lines. Comparing differentially expressed genes that were upregulated in NSD2 High cell lines to those upregulated in NSD2 High patients revealed significant overlap whereas, as expected, cell line NSD2 High downregulated genes with patient NSD2 High downregulated genes did not (Additional file 1: Fig. S7c). We also show insignificant overlap of genes upregulated upon relapse in 9 matched D/R patient pairs with no NSD2 mutation (NSD2 Low to NSD2 Low) [26] and our cell line NSD2-High upregulated genes with a B to A compartment switch or shift (Additional file 1: Fig. S7c). Importantly, 37.94% of upregulated genes in patients overlapped with a BA switch or shift in cell lines (Additional file 1: Fig. S7d). Comparing those cell line NSD2 High genes upregulated and mediated by compartment switches and shifts to those upregulated in NSD2 High patients also revealed significant overlap (Fig. 6d). Additionally, differentially expressed genes in patients were found to be enriched in B to A changes compared with A to B changes (Fig. 6e). Upregulated genes in NSD2 High patients were also found to correlate with B to A changes whereas downregulated genes did not correlate with A to B changes as we had observed in cell lines (Fig. 6f). Lastly, we performed pathway enrichment analysis with those genes upregulated in NSD2 High patients that overlapped cell line compartment changes (Fig. 6g). Using this patient data, we identified numerous pathways that have been shown previously with NSD2 cell line models, such as calcium signaling, Rap1 signaling, and cell adhesion pathways. Interestingly, some of these pathways were identified because of shared compartment/gene expression changes across the cell lines, such as NEO1 for cell adhesion, while others were found in only 1 or 2 of the lines, such as FGF5 or FGFR2 associated with Rap1 and calcium signaling. This data demonstrates changes in compartments unique to individual cell lines converge on pathways important for clonal evolution and drug resistance while others are shared across cell lines.

### NSD2 EK-mediated epigenetic changes correlate with 3D genome changes

**Fig. 7:**
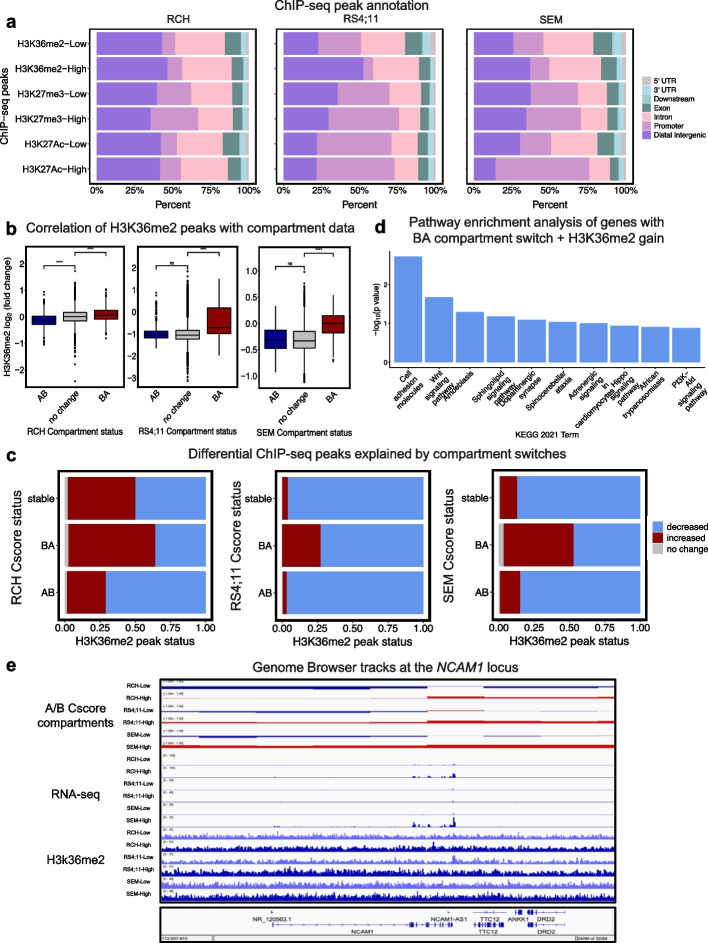
NSD2 EK-mediated epigenetic changes correlate with 3D genome changes. **a** Bar plots showing distribution of annotated ChiP-seq peaks for each epigenetic mark for NSD2-Low and NSD2-High in each cell line. **b** Correlation boxplots of H3K36me2 ChIP-seq and cscore compartment switches. **c** Association barplots showing fraction of differential H3K36me2 chip-seq peaks (decreased, increased, and no change) at cscore compartment switches (AB, BA, and stable). **d** KEGG 2021 pathway enrichment analysis of genes upregulated from NSD2-Low to NSD2-High that overlap with B to A compartment switches and increased H3K36me2 peaks from all three cell lines. **e** IGV genome browser tracks showing an example of concordant changes in A/B compartments, H3K36me2, and gene expression from NSD2 Low to NSD2 High at the *NCAM1* gene locus in all three cell lines

We previously performed ChIP-seq on the RS4;11 NSD2 Low and NSD2 High cell lines [5]. For this work, we additionally performed ChIP-seq on the RCH and SEM NSD2 Low and NSD2 High cell lines for H3K36me2, H3K27me3, and H3K27ac epigenetic marks. As noted previously, H3K36me2 distribution shifted from predominantly promoter regions to intergenic regions in all three cell lines, most dramatically in the RS4;11 cell line (Fig. 7a). Conversely, H3K27me3 peaks shifted from intergenic regions to promoter regions (Fig. 7a). Notably, H3K27ac was found to be enriched at promoter regions with little difference in distribution (Fig. 7a).

To examine the relationship between alterations in the distribution of H3K36me2 and A/B compartment switches, we categorized the A/B compartment switches into those that are AB, BA, or stable and assessed changes in H3K36me2. H3K36me2 peaks positively correlated with BA switches more significantly than with AB switches in each of the cell lines (Fig. 7b). We also identified that B to A compartment switches were associated with increased H3K36me2 (Fig. 7c). This data suggests that expansion of H3K36me2 as a result of NSD2 EK provides a more open chromatin landscape.

From previous work, we know that NSD2 regulates H3K36me2; therefore, we can reasonably argue that the H3K36me2-related gene expression changes are due to NSD2. Under this assumption, we calculated the percentage of gene changes that can be attributed to both B to A compartment switches/shifts and increases in H3K36me2 (Additional file 1: Fig. S9a). Approximately 16–30% of genes that were upregulated were within a decompacted compartment, while ~16% presented with increased H3K36me2. For the differentially expressed genes that cannot be attributed to NSD2-related compartment switches, we believe these might be the result of indirect effects.

Next, to examine the relationship between alterations in the distribution of H3K36me2 and intra-TAD activity, we categorized the intra-TAD activity changes into those that are decreased, increased, or stable and assessed changes in H3K36me2. Although there were few intra-TAD activity changes, we observed that increased intra-TAD activity predominantly consisted of increased H3K36me2 and that decreased intra-TAD activity predominantly consisted of decreased H3K36me2 (Additional file 1: Fig. S9b).

Lastly, to better understand the impact these changes have on downstream signaling, we performed pathway enrichment analysis for those differentially expressed genes that were associated with a compartment switch from B to A and an increase in H3K36me2 in any of the cell lines (Fig. 7d). Wnt signaling and cell adhesion were identified as the two most significant pathways. Interestingly, we observed a B to A compartment switch, increased H3K36me2 peaks, and increased gene expression at the *NCAM1* gene locus in all three cell lines (Fig. 7e), one of the genes that is related to cell adhesion pathways. Importantly, while cell adhesion was identified in several of our pathway analyses, some genes were impacted by both 3D change and epigenetic modifications such as *NCAM1*, while *NEO1* showed a compartment change but no change in H3K36me2. This highlights the convergence of NSD2 EK-mediated epigenetic modifications and 3D genome organization on downstream signaling.

## Discussion

Our previous work demonstrated enrichment of the NSD2 EK mutation in pediatric B-ALL patients at relapse and confirmed its role in cell proliferation, clonogenicity, and therapy resistance [5]. The lack of effective agents to inhibit the enzymatic activity of NSD2 has hampered targeted therapy for NSD2 rearranged multiple myeloma and NSD2 E1099K relapsed ALL. Moreover, we and others have demonstrated NSD2 EK activates a transcriptional program that is highly cell-context dependent, possibly endowing cancer cells with a broad repertoire of pathways needed to navigate the selective pressures of therapy. However, the lack of common genes impacted by NSD2 EK makes a single targeted strategy difficult. Herein we show that NSD2 EK plays an essential role in reorganizing the 3D genome through a striking reliance on compartment reorganization and uncover a convergence on pathways vital to cancer progression, indicating the translational relevance of our findings.

Hi-C analysis of NSD2 Low and NSD2 High cell lines revealed significant compartment changes (26.06%). This is consistent with a multiple myeloma model in which 23.00% of compartment changes were identified [15]. To put this finding into context, only 3.00–5.00% compartment shifts were identified in various stages of B-cell differentiation including pre-B to pro-B and pro-B to immature-B (unpublished data). Consistent with NSD2 EK’s role in increased H3K36me2 methyltransferase activity, we observed significantly more B to A compartment switches and shifts, compared with A to B switches and shifts. This is in opposition to the multiple myeloma model in which low expressing vs high expressing cells showed comparable A to B and B to A switches [15].

Hi-C analysis also revealed shared compartment switches across the three cell lines including 219 shared switches from B to A and 50 from A to B (Fig. 1e). Additionally, 22.40% (932/4,160) of B to A switches and 16.20% (269/1,661) A to B switches were shared by at least 2 cell lines. Previous work demonstrating variability in the phenotypic impact of NSD2 EK on proliferation, clonal growth, and sensitivity to cytotoxic chemotherapeutic agents attributed this diversity to cell context. Here, we highlight the existence of a common core of decompacting loci, attributed to the NSD2 EK mutation, that can explain previously described universal features, such as proliferation, as well as serve as targets for therapeutic intervention. While we used *NEO1* as an example, several other genes known to have roles in cancer proliferation and various pathway cascades were identified within these shared compartments, including *EPS8, FAM92A, IRS1*, and *SETBP1*.

Importantly, those compartments that switched to a less compact state showed increased H3K36me2 and decreased H3K27me3 epigenetic marks, suggesting that NSD2 EK plays a prominent role in chromatin decompaction through enrichment of H3K36me2 epigenetic marks. The expansion of H3K36me2 past gene bodies into more distal intergenic regions was also revealed. These data suggest that NSD2 EK creates a more open, active chromatin environment by spreading the H3K36me2 mark perhaps endowing cells with an increased repertoire of genes to respond effectively to the selective pressures of treatment.

Furthermore, compartment changes and epigenetic changes significantly correlated with genes that were upregulated due to NSD2 EK. For those compartments that became compact (A to B), we did not see a clear association with decreased gene expression. Whether alterations in the epigenetic landscape can impact chromosome organization in a manner that corresponds to alterations in gene expression has been a long-standing question in the field. As Lhoumaud et al. described in multiple myeloma, our data also highlight a bidirectional relationship between 2D and 3D chromatin structure in gene regulation [15]. We propose a model in which the NSD2 EK mutation directly results in the expansion of the H3K36me2 landscape, and this is associated with genome-wide chromatin decompaction as well as concordant changes in gene expression. However, we did observe dysregulation of gene expression in the absence of any 3D or epigenetic change, suggestive of possible indirect or downstream effects of NSD2.

Although EK-mediated changes in transcriptional output as well as chromatin accessibility differed dramatically between cell lines, pathway analysis on differential genes with compartments that changed from B to A demonstrated convergence on similar pathways, such as cell adhesion, Rap1, and calcium-related pathways. Interestingly, these pathways have also come up in our previous analysis with RNA-seq data alone as well as in other models [7]. Cell adhesion genes were identified in BA compartment switches associated with H3K36me2 gain and in clinical samples with NSD2 EK at relapse. Swaroop et al. demonstrated that the NSD2 EK mutation was associated with increased adhesion to stromal cells as well as increased migratory properties [7]. The *NEO1* gene was upregulated by a compartment shift in all three cell lines as well as in the patient samples and encodes neogenin-1 a cell surface receptor implicated in a variety of cell processes such as cell survival, angiogenesis, and migration. Neogenin has been associated with cell proliferation and motility in a number of solid tumors but not in hematological malignancies. Recent work indicates a key role in maintaining hematopoietic stem cell quiescence and long-term self-renewal [30]. Likewise, the small GTPase Rap1 is impacted by intracellular calcium in some circumstances, two pathways we also identified, and is known to stimulate integrin activation [31]. *FGF5* and *GNAQ* were two of the genes implicated in Rap1 and calcium signaling in the patient samples but were found to be specific to either RS4;11 or RCH cells, respectively [32–34]. FGF5 has been shown to affect proliferation and migration in melanoma and glioblastoma multiforme models while GNAQ activates PKC, FAK, and ERK signaling cascades to promote proliferation and cell survival in uveal melanoma. These examples highlight how the underlying differences in the 3D organization leads to the activation of various genes that ultimately provide a clonal advantage. In addition, WNT and MAPK (Ras-related) pathways were identified in two out of the three lines examined and have also previously been linked to NSD2 in the multiple myeloma model [15]. The identification of common downstream targets among preclinical models and patient samples has important clinical implications for possible future therapeutic interventions.

By examining the regions where we observed compartment decompaction, increased accessibility, and upregulation of gene expression, we identified enrichment of motifs linked to stem cell functionality, including NANOG, SUZ12, SOX2, and MEIS1. In the B-cell lymphoma model, Yusufova et al. also demonstrated that many differentially expressed genes were linked to stem cell functionality as well as enriched for various stem cell signature-related pathways [16]. This data suggests a potential core transcription factor network that may be dependent on compartmentalization.

In contrast to the identification of widespread compartment switches upon, we identified very few changes in intra-TAD activity. This data suggests that NSD2 EK-mediated changes are primarily linked to changes in compartmentalization. Furthermore, this supports the notion that TADs and compartments are established by separate, competing mechanisms. Our findings are also in agreement with a recent paper demonstrating H1 loss-of-function mutations in B-cell lymphomas leading to chromatin decompaction. Few TAD changes were observed and H1 loss led to decompaction through increased H3K36me2, again supporting the idea of NSD2 playing a role in chromatin decompaction [16]. Although few TAD activity changes were identified, decreasing TAD activity was predominantly associated with A to B compartment switches and increasing TADs predominantly associated with B to A switches suggesting that TADs might be susceptible to patterns established by compartments.

## Conclusions

Here we have identified a link between NSD2 EK-mediated epigenetic changes and dysregulation of higher-order genomic architecture in B-ALL. Our study revealed NSD2 EK’s prominent role in chromatin decompaction through enrichment of H3K36me2 epigenetic marks. We also demonstrated NSD2 EK’s remarkable reliance on compartment reorganization over TAD activity. Despite transcriptional and chromatin accessibility heterogeneity across the three cell lines, this study highlights the existence of a common core of decompacting loci that can explain previously described universal features, such as proliferation, as well as serve as targets for therapeutic intervention. Ultimately, this study offers a novel mechanism by which NSD2 EK creates a more open, active chromatin state through decompaction allowing for diverse transcriptional reprogramming in response to selective pressures associated with treatment.

## Methods

### Experimental procedures

Cell lines, RNA-seq, ATAC-seq, and ChIP-seq methods published previously [5]. The B-lineage leukemia cell lines RS4;11, RCH-ACV, and SEM were used for the purposes of this study. RS4;11 was acquired from ATCC (Manassas, VA), RCH-ACV was acquired from DSMZ (Braunschweig, Germany), and SEM was kindly gifted by Jun Yang, St. Jude Children’s Hospital. Each leukemia cell line was validated by short tandem repeat analysis through ATCC except for RCH-ACV which was purchased from DSMZ directly. DSMZ routinely verifies cell lines and provides authentication information with each order. Cell lines were routinely monitored for mycoplasma contamination by PCR using ATCC Universal Mycoplasma Detection Kit (20–1012K). NSD2 Low cell lines were generated in these three B-ALL cell lines because they naturally harbor a heterozygous NSD2 EK mutation. More specifically, a short-hairpin RNA (shRNA: GGAAACTACTCTCGATTTATG) was used to knockdown NSD2 targeting the NSD2 Type II and NSD2 RE-IIBP isoforms both of which contain the SET domain.

### Primary patient sample experimental procedures

Cryopreserved PBMC samples from Children’s Oncology Group (COG) B-ALL study AALL-1331 were obtained from the COG Biobank. All subjects provided consent for banking and future research use of these specimens in accordance with the regulations of the institutional review boards of all participating institutions. Samples were resuspended in TRIzol reagent (Life Technologies) then processed using 5prime Phase Lock Gel Heavy tubes (Quantabio) following the manufacturer’s instructions. Following precipitation, the RNA was quantitated using the Qubit RNA BR assay kit (Life Technologies) and evaluated for quality using the Agilent 2100 Bioanalyzer with the RNA nano chip (Agilent). The KAPA RNA HyperPrep Kit with RiboErase (HMR) (Roche) with 100–200ng of total RNA was used for library construction with either Nextflex-96 RNA barcode adapters (BIOO Scientific, Illumina HiSeq 4000 platform), or IDT for Illumina unique dual indices adaptors (Illumina, Novaseq 6000 platform) and 12 PCR cycles of library amplification. Libraries were evaluated on the Bioanalyzer 2100 using the DNA 1000 chip. Libraries were sequenced using either the HiSeq 4000 (Illumina) or NovaSeq 6000 (Illumina) paired-end 2X150 cycle protocol.

### Hi-C experimental procedures

Five million cells from actively growing NSD2 High and Low cell lines were crosslinked with 2% PFA and quenched with glycine. Following crosslinking, cells were lysed and DNA digested and proximally ligated following Arima Genomics’ Hi-C kit User Guide for Mammalian Cell Lines by the NYU Langone Genome Technology Center. Hi-C libraries were generated using Arima-HiC Kit along with Swift 2S Plus Kit and Swift Indexing Kit following Arima-HiC Kit Library Preparation using Swift Biosciences(R) Accel-NGS(R) 2S Plus DNA Library Kit User Guide. Libraries were sequenced using Illumina’s NovaSeq 6000.

### Computational analysis

#### Cell line RNA-seq analysis

Cell line RNA-seq fastq files were processed in triplicates using the route “rna-star” and “rna-star-groups-dge” from the Slide-n-Seq (sns) pipeline: https://igordot.github.io/sns/. Processing steps include alignment of paired-end reads to the human reference genome (hg19) using the STAR aligner with default parameters [35]. Counts were obtained using featureCounts [36]. Bigwig tracks were obtained for visualization on individual samples using deeptools (v3.1.0) [37]. Downstream analysis including normalization and differential expression analysis was performed using DESeq2 [38]. Genes were categorized as differentially expressed if abs(L2FC > 0.32, *p*-value < .05). Pathway analysis was performed using enrichR [39].

#### Primary patient sample RNA-seq analysis

COG RNA-seq count data was provided to us by Dr. Xiaotu Ma from the Department of Computational Biology at St. Jude Children’s Research Hospital. Count data was generated using HTSeq [40]. Downstream analysis including normalization and differential expression analysis was performed using DESeq2 [38]. Genes were categorized as differentially expressed if abs(L2FC > 0.32, *p*-value < .05). Pathway analysis was performed using enrichR [39].

#### ATAC-seq analysis

Cell line ATAC-seq fastq files were processed in two replicates using the route “atac” from the Slide-n-Seq (sns) pipeline: https://igordot.github.io/sns/. Processing steps included aligning paired-end reads to the human reference genome(hg19) with Bowtie2 (v2.3.4.1) [41]. Reads with a mapping quality <30 were removed. Duplicated reads were removed using Sambamba (v0.6.8) [42]. Remaining reads were analyzed by applying the peak-calling algorithm MACS2 (v2.1.1) [43]. Bigwig tracks were obtained for visualization on individual samples using deeptools (v3.1.0) [37]. Differential ATAC-seq analysis was performed using DiffBind [44]. Nearest genes were annotated using ChIPseeker [45]. Enrichment analysis was performed using Bioconductor package LOLA (Locus overlap analysis or enrichment of genomic ranges; R package version 1.24.0) with RStudio (v3.6.1) [46]. Enrichment analysis of genomic regions sets was performed using Bioconductor package LOLA (Locus overlap analysis or enrichment of genomic ranges; R package version 1.24.0) with RStudio (v3.6.1) with the hg19 LOLA core database [46]. LOLA core database is curated from many sources including TF binding sites from Encode and epigenome databases from Cistrome.

#### ChIP-seq analysis

Processing of cell line ChIP-seq fastq files was performed by the Carroll lab’s previous bioinformatician, Gunjan Sethia, as previously described [5]. Cell line ChIP-seq data was processed with three replicates. Paired-end reads were aligned to the human reference genome (hg19) with Bowtie2 (v2.3.4.1) [41]. Reads with low mapping quality <30 were discarded using Trimmomatic (v0.33; ref. 23) [47]. Due to the broad/diffuse peaks created by H3K36me2 and H3K27me3, peaks for these marks were called by SICER that uses a cluster enrichment–based analysis [48]. H3K27ac and H3K9ac peaks were called using MACS2 (–broad; ref. 30) [43]. Bigwig tracks were obtained for visualization on individual samples using deeptools (v3.1.0) [37]. Differential ChIP-seq analysis was performed using DiffBind [44]. Peaks were categorized as differentially accessible if abs(L2FC > 1.0, FDR < .01). Nearest genes were annotated using ChIPseeker [45].

#### Hi-C analysis

Raw Hi-C sequencing data was processed with the hic-bench platform [19]. Cell line Hi-C data was processed as single replicates. Data was aligned against the human reference genome(GRCh37/hg19) with bowtie2(version 2.3.1) [41]. The reads used for downstream analyses were filtered for by the GenomicTools tools-hic filter command in the hic-bench platform using default parameters. The GenomicTools tools-hic filter command discards reads including multi-mapped reads (“multihit”), read-pairs with only one mappable read (“single sided”), duplicated read-pairs (“ds.duplicate”), read-pairs with a low mapping quality of MAPQ < 20, read-pairs resulting from self-ligated fragments (together called “ds.filtered”), and short range interactions resulting from read-pairs aligning within 25kb (“ds.too.short”). Downstream analysis is performed with the accepted intra-chromosomal read- pairs (“ds.accepted intra”). The number of accepted intra-chromosomal read-pairs varied between ~40 and ~140 million for all samples (Chapter 1; Fig. 2). Hi-C interaction matrices were generated for each chromosome separately by the hic-bench platform at 40kb resolution. Filtered read counts were normalized by iterative correction and eigenvector decomposition (ICE) [49]. To account for variances in read counts of more distant loci, distance normalization for each chromosome matrix was performed.

### Hi-C contact matrix visualization

To visualize the Hi-C contact matrix for the PRDM8/FGF5 locus, ICE normalized Hi-C contact matrices for the corresponding chromosome was loaded and normalized by the total number of intra-chromosomal interactions for NSD2 Low and NSD2 High samples. The log2FC Hi-C contact matrix was produced by applying the log2 function on the division product of the NSD2 High Hi-C table by the NSD2 Low Hi-C table.

### A/B compartments analysis

A/B compartment analysis was performed using Cscore tool. The Cscore tool algorithm was used to assign active (A) and inactive (B) compartments [20]. Cscore assumes that each genomic window, *i*, has a chance *P*_*i*_ to be in the A compartment. By defining Cscore as *C*_*i*_=2*P*_*i*_−1⁠, which ranges between −1 and 1, Cscore deduces a log-likelihood function where *n*_*ij*_ is the observed number of contacts, *d*_*ij*_ is the distance along the genome, *H*(*d*_*ij*_) is the scaling factor accounting for the decrease of interactions at longer genomic distance, and *B*_*i*_ and *B*_*j*_ are the bias factors from Hi-C experiments. A maximum-likelihood estimation was performed for the model parameters B, C, and H using an iterative algorithm:

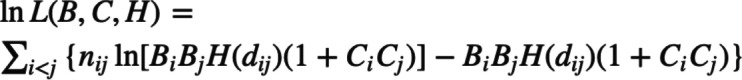



For a bin to be considered a switch from A to B or from B to A, the compartment score sign had to flip in sample 2 (NSD2 High) when compared with the reference sample (sample 1 or NSD2 Low). The absolute difference between the compartment scores had to be higher than the cutoff (default: 1.2). The difference is computed as a relative delta:

delta = (*Y − X*) / abs(*Y*) #delta value calculation

*X* = compartment score of bin in sample 1 (reference)

*Y* = compartment score of bin in sample 2

For the compartment switch analysis, percent switches in compartments was calculated by quantifying the sum of the total number of bins that switched from A to B and B to A divided by the total number of compartment bins (switching bins + stable bins). For compartment switch and shift analysis, bins were classified as A to B or B to A switches if there was a switch in the compartment Cscore sign. Bins were classified as B to more B, B to less B, A to more A, or A to less A if there was not a switch in the compartment Cscore sign but the delta exceeded a cutoff of delta > 0.2. Additionally, percent switches in compartments was calculated by quantifying the sum of the total number of bins that switched from B to more B, B to less B, A to more A, A to less A, A to B, and B to A divided by the total number of compartment bins (switching bins + shifting bins + stable bins).

### Intra-TAD activity analysis

Intra-TAD activity was assessed using the “domains” and “domains-diff” steps within the hic-bench platform [19]. The “domains” step uses the hic-ratio algorithm for TAD calling developed within hic-bench by previous Tsirigos lab member Haris Lazaris in which the average of the normalized interaction scores is calculated for all interactions taking place within a particular TAD. The “domains-diff” step assesses TAD interactivity alterations and was developed by previous lab members Sofia Nomikou and Andreas Kloetgen [17, 19]. To identify TADs with differential TAD interactivity between NSD2 Low and High cell lines, we used the TADs identified in the NDS2 Low sample as a reference to identify common TADs. Once intra-TAD values are obtained from the Hi-C data for NSD2 Low and NSD2 High cell lines, a Wilcoxon two-sided rank sum non-parametric test is performed to determine the *p*-values for each TAD. Multiple testing is used to correct these *p*-values by adjusting to the total number of TADs. Lastly, the log2 fold change (log2FC) of intra-TAD activity value is calculated between the samples. Intra-TAD activity alterations were categorized as significant if abs(L2FC > 0.25) and FDR < 0.01.

### Integration of compartment data with other datasets

To show the correlation between the compartment changes and the various sequencing datasets, we first calculated the peak intensity fold change or gene expression fold change between NSD2 Low and High cell lines for peaks obtained from H3K36me2, H3K27me3, H3K27ac, ATAC-seq, and the genes obtained from RNA-seq through a differential analysis as described above. Differential peaks and genes were then classified as increased, decreased, or stable according to thresholds described above and then mapped to the AB, BA, or stable regions using the “bedtools intersect” command [50, 51]. Correlation between the compartment changes and the various sequencing datasets were then shown with boxplots or bar plots. Statistical significance was assessed using a paired two-sample *t*-test.

## Supplementary Information

**Additional file 1: Figure S1a.** Hi-C million read counts (left) and read percentage (right) per cell line. **Figure S2a.** Venn diagram showing overlap of differentially expressed genes (abs(L2FC) > 0.32, *p*-value < 0.05) between three B-ALL cell lines upon knockdown, decreased and increased genes (left and right respectively). b. Volcano plots demonstrating differentially expressed genes (abs(L2FC) > 0.32, *p*-value < 0.05) highlighted by compartment switch or shift (A to more A, B to A, B to less B, or stable). c. Scatterplot demonstrating compartments colored by concordance score (percentage of genes with that compartment switch or shift that change in the same direction). **Figure S3a.** Rate of concordance calculations represent the amount of upregulated and downregulated genes (abs(L2FC) > 0.32, *p*-value < 0.05) that can be explained by compartment switches and shifts. **Figure S4a.** Barplot showing number of subcompartment calls with SNIPER for each cell line at NSD2 Low and NSD2 High. b. Barplots presenting fraction of Cscore compartment calls (A or B) overlapping SNIPER subcompartment calls (A1, A2, B1, B2, and B3) c. Barplot showing number of subcompartment calls with SNIPER for each cell line at NSD2 Low and NSD2 High excluding B1 subcompartment calls. d. Barplot presenting number of subcompartment switches from A to B and from B to A from NS2D Low to NSD2 High in each of the cell lines. e. Barplots presenting fraction of SNIPER subcompartment calls overlapping cscore compartment switches and shifts. **Figure S5a.** Correlation boxplots of SNIPER subcompartments and gene expression changes (abs(L2FC) > 0.32, *p*-value 0.05) for each cell line from NSD2 Low to NSD2 High. b. Volcano plots demonstrate differentially expressed genes (abs(L2FC) > 0.32, *p*-value < 0.05) highlighted by subcompartment switches and shifts. **Figure S6a.** PCA of ATAC-seq peaks for each cell line identifies three distinct clusters which are cell line-specific. b. Heat map representation of ATAC-seq results generated with DiffBind. c. Venn Diagram showing overlap of differentially accessible regions between three B-ALL cell lines decreased and increased accessibility (left and right respectively; (FDR < 0.01, abs(log2(fold change)) > 1). d. Example of B to A compartment shift shared by all three cell lines at the *NEO1* locus showing concordance with gene expression and chromatin accessibility. e. Example of B to A compartment shift specific to RS4;11 cell line at the *PRDM8* locus showing concordance with gene expression and chromatin accessibility. **Figure S7a.** Heatmap representation of mean intra-TAD activity per cell line. b. Bar plot showing number of TAD activity changes per cell line (abs(L2FC) > 0.25, FDR < 0.01). c. Venn diagrams demonstrating significant overlap of patient NSD2-High upregulated genes and cell line NSD2-High upregulated genes and insignificant overlap of patient NSD2-High downregulated genes and cell line NSD2-High upregulated genes (one-tailed Fisher’s exact test). The last row presents insignificant overlap of genes upregulated in 9 matched D/R patient pairs with no NSD2 mutation (NSD2 Low) and cell line NSD2-High upregulated genes with a B to A compartment switch or shift. d. Table presenting percentage of patient NSD2-High upregulated genes overlapping a compartment switch/shift from any of the three cell lines. **Figure S8a.** Table presents patient information associated with the three matched diagnosis-relapse pairs acquired from COG. **Figure S9a.** Rate of concordance calculations present the number of genes upregulated in expression, increased in H3K36me2 ChIP-seq peaks, and B to A compartment shifts and switches for the RCH, RS4;11, and SEM cell lines. b. Association bar plots showing fraction of differential H3K36me2 chip-seq peaks (decreased, increased, and stable) that overlap changes in intra-TAD activity (increased, decreased, and stable TADs).

**Additional file 2.** stable_genomic_loci.bed LOLA Analysis File of stable ATAC-seq peaks.

**Additional file 3.** AB_genomic_loci.bed LOLA Analysis File of ATAC-seq peaks with A to B compartment switch.

**Additional file 4.** BA_genomic_loci.bed LOLA Analysis File of ATAC-seq peaks with B to A compartment switch.

**Additional file 5.** LOLA_enrichment_results.tsv LOLA Analysis Results File.

**Additional file 6.** shared_comp_genes_down.csv Expression of decreased genes with shared AB compartment switches.

**Additional file 7.** shared_comp_genes_up.csv Expression of increased genes with shared BA compartment switches.

**Additional file 8.** TAD_increased.csv Gained TAD activity due to NSD2 coordinates.

**Additional file 9.** TAD_decreased.csv Lost TAD activity due to NSD2 coordinates.

**Additional file 10.** TAD_expression_cscore_AB.csv Concordant lost TAD activity, decreased gene expression, and AB compartment switch.

**Additional file 11.** TAD_expression_cscore_BA.csv Concordant gained TAD activity, increased gene expression, and BA compartment switch.

**Additional file 12.** TAD_expression_down.csv Concordant lost TAD activity and decreased gene expression.

**Additional file 13.** TAD_expression_up.csv Concordant gained TAD activity and increased gene expression.

**Additional file 14.** Review history.

## Data Availability

The cell line Hi-C dataset supporting the conclusions of this article is available in the National Center for Biotechnology Information Gene Expression Omnibus (GEO Series accession number GSE199754: https://www.ncbi.nlm.nih.gov/geo/query/acc.cgi?acc=GSE199754) [52]. The COG patient sample dataset (RNA-seq) supporting the conclusions of this article is available in the National Center for Biotechnology Information database of genotypes and phenotypes (dbGaP accession number phs003195.v1.p1;https://www.ncbi.nlm.nih.gov/projects/gap/cgi-bin/study.cgi?study_id=phs003195.v1.p1) [53]. The previously generated cell line dataset (ChIP-seq, ATAC-seq, and RNA-seq) supporting the conclusions of this article is available in the National Center for Biotechnology Information Gene Expression Omnibus (GEO Series accession number GSE149159; https://www.ncbi.nlm.nih.gov/geo/query/acc.cgi?acc=GSE149159) [5, 54].
