# Supplementary material for: NSD2 E1099K drives relapse in pediatric acute lymphoblastic leukemia by disrupting 3D chromatin organization
Source: Genome Biol. 2023 Apr 4;24:64. doi: 10.1186/s13059-023-02905-0 (PMC10071675; doi:10.1186/s13059-023-02905-0)
Supplement: Supplementary file 1 — Additional file 1: Figure S1a. Hi-C million read counts (left) and read percentage (right) per cell line. Figure S2a. Venn diagram showing overlap of differentially expressed genes (abs(L2FC) > 0.32, p-value < 0.05) between three B-ALL cell lines upon knockdown, decreased and increased genes (left and right respectively). b. Volcano plots demonstrating differentially expressed genes (abs(L2FC) > 0.32, p-value < 0.05) highlighted by compartment switch or shift (A to more A, B to A, B to less B, or stable). c. Scatterplot demonstrating compartments colored by concordance score (percentage of genes with that compartment switch or shift that change in the same direction). Figure S3a. Rate of concordance calculations represent the amount of upregulated and downregulated genes (abs(L2FC) > 0.32, p-value < 0.05) that can be explained by compartment switches and shifts. Figure S4a. Barplot showing number of subcompartment calls with SNIPER for each cell line at NSD2 Low and NSD2 High. b. Barplots presenting fraction of Cscore compartment calls (A or B) overlapping SNIPER subcompartment calls (A1, A2, B1, B2, and B3) c. Barplot showing number of subcompartment calls with SNIPER for each cell line at NSD2 Low and NSD2 High excluding B1 subcompartment calls. d. Barplot presenting number of subcompartment switches from A to B and from B to A from NS2D Low to NSD2 High in each of the cell lines. e. Barplots presenting fraction of SNIPER subcompartment calls overlapping cscore compartment switches and shifts. Figure S5a. Correlation boxplots of SNIPER subcompartments and gene expression changes (abs(L2FC) > 0.32, p-value 0.05) for each cell line from NSD2 Low to NSD2 High. b. Volcano plots demonstrate differentially expressed genes (abs(L2FC) > 0.32, p-value < 0.05) highlighted by subcompartment switches and shifts. Figure S6a. PCA of ATAC-seq peaks for each cell line identifies three distinct clusters which are cell line-specific. b. Heat map representation of ATAC-seq results g [file 13059_2023_2905_MOESM1_ESM.pptx]

## Slide 1
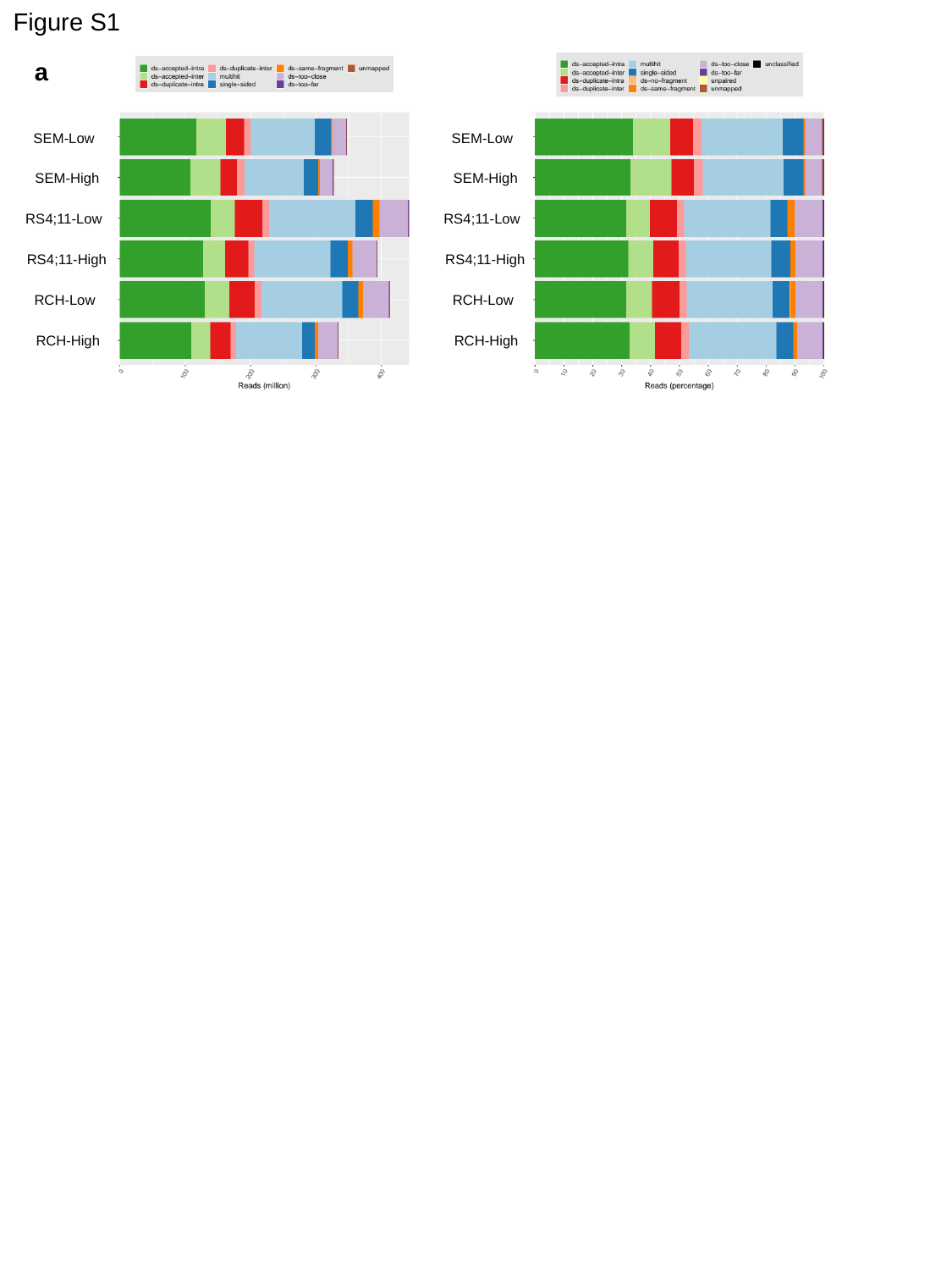

Figure S1
a
SEM-Low
SEM-High
RS4;11-Low
RS4;11-High
RCH-Low
RCH-High
SEM-Low
SEM-High
RS4;11-Low
RS4;11-High
RCH-Low
RCH-High

## Slide 2
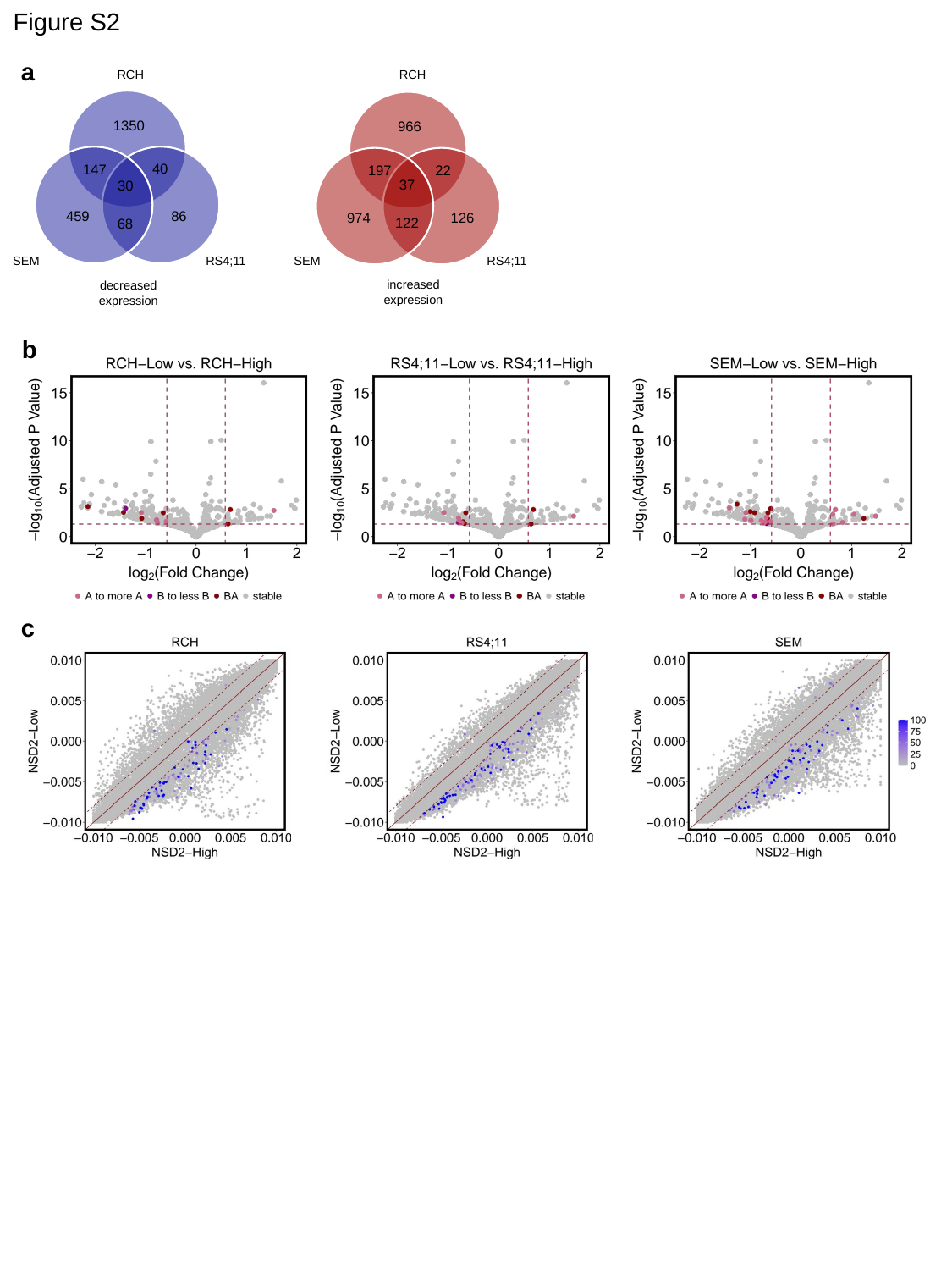

Figure S2
a
RCH
RCH
1350
40
147
30
459
86
68
966
22
197
37
974
126
122
SEM
RS4;11
SEM
RS4;11
increased
expression
decreased
expression
b
c

## Slide 3
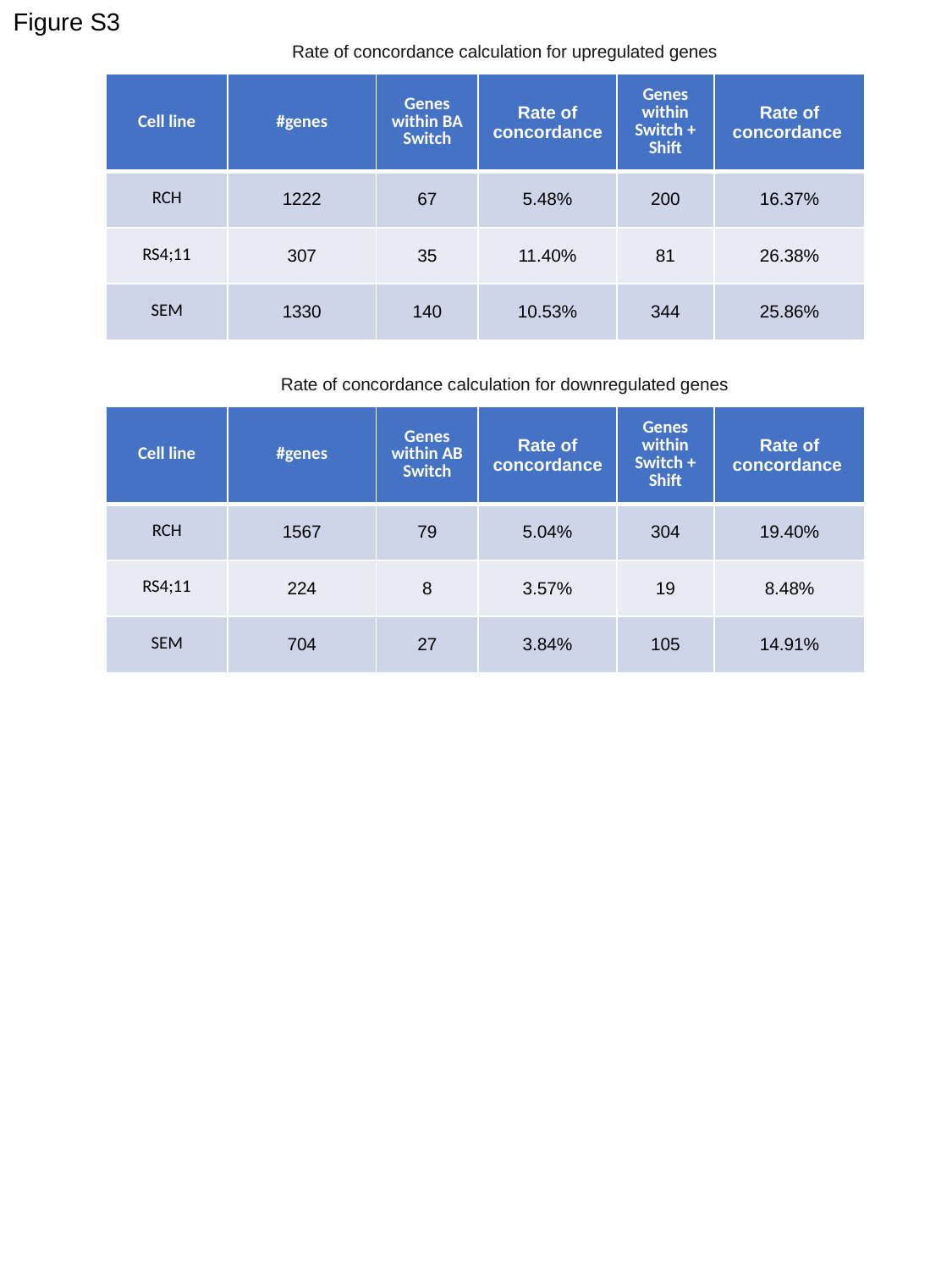

Figure S3
Rate of concordance calculation for upregulated genes
| Cell line | #genes | Genes within BA Switch | Rate of concordance | Genes within Switch + Shift | Rate of concordance |
| --- | --- | --- | --- | --- | --- |
| RCH | 1222 | 67 | 5.48% | 200 | 16.37% |
| RS4;11 | 307 | 35 | 11.40% | 81 | 26.38% |
| SEM | 1330 | 140 | 10.53% | 344 | 25.86% |
Rate of concordance calculation for downregulated genes
| Cell line | #genes | Genes within AB Switch | Rate of concordance | Genes within Switch + Shift | Rate of concordance |
| --- | --- | --- | --- | --- | --- |
| RCH | 1567 | 79 | 5.04% | 304 | 19.40% |
| RS4;11 | 224 | 8 | 3.57% | 19 | 8.48% |
| SEM | 704 | 27 | 3.84% | 105 | 14.91% |

## Slide 4
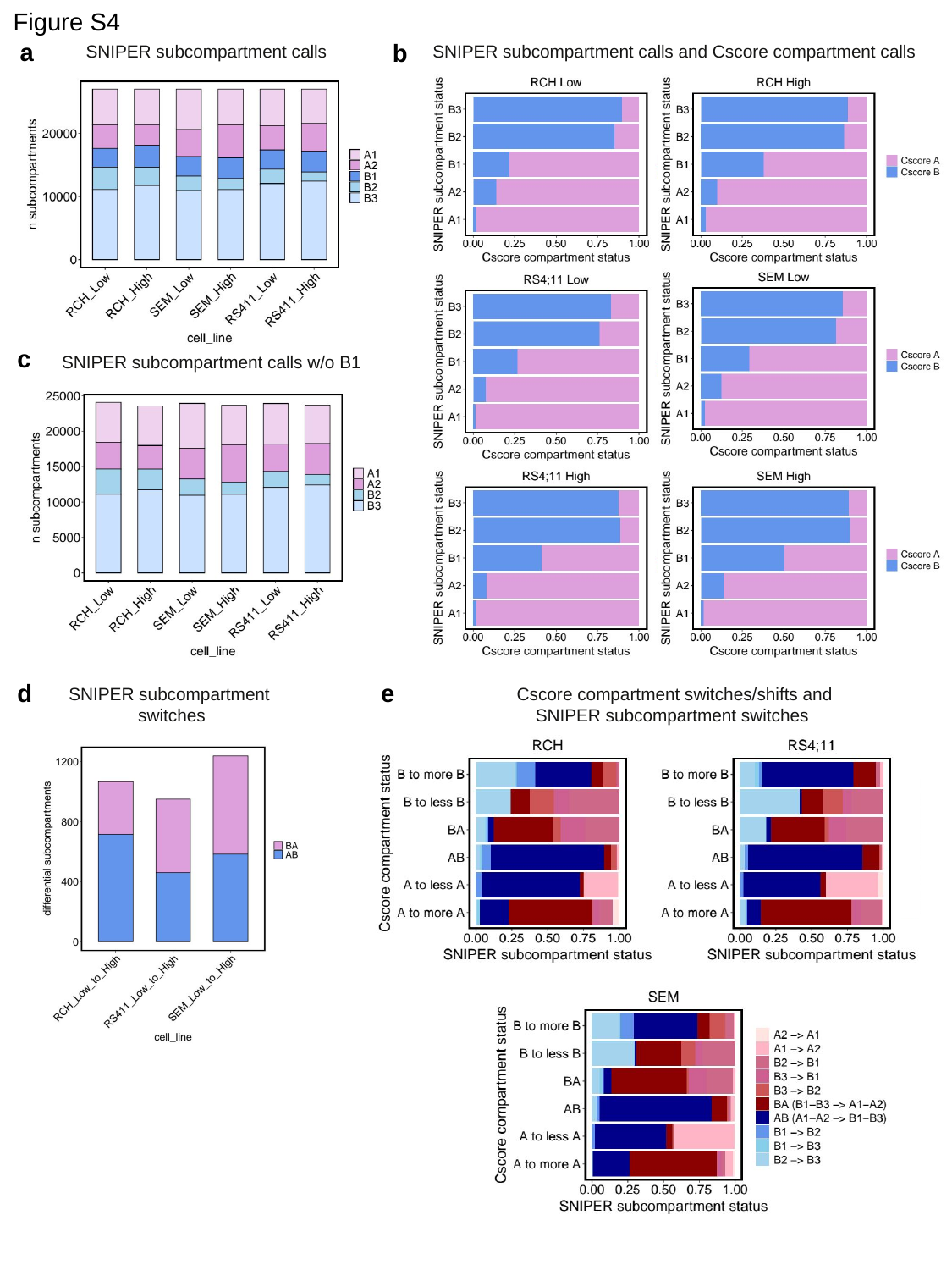

Figure S4
a
b
SNIPER subcompartment calls
SNIPER subcompartment calls and Cscore compartment calls
c
SNIPER subcompartment calls w/o B1
e
d
SNIPER subcompartment
switches
Cscore compartment switches/shifts and SNIPER subcompartment switches

## Slide 5
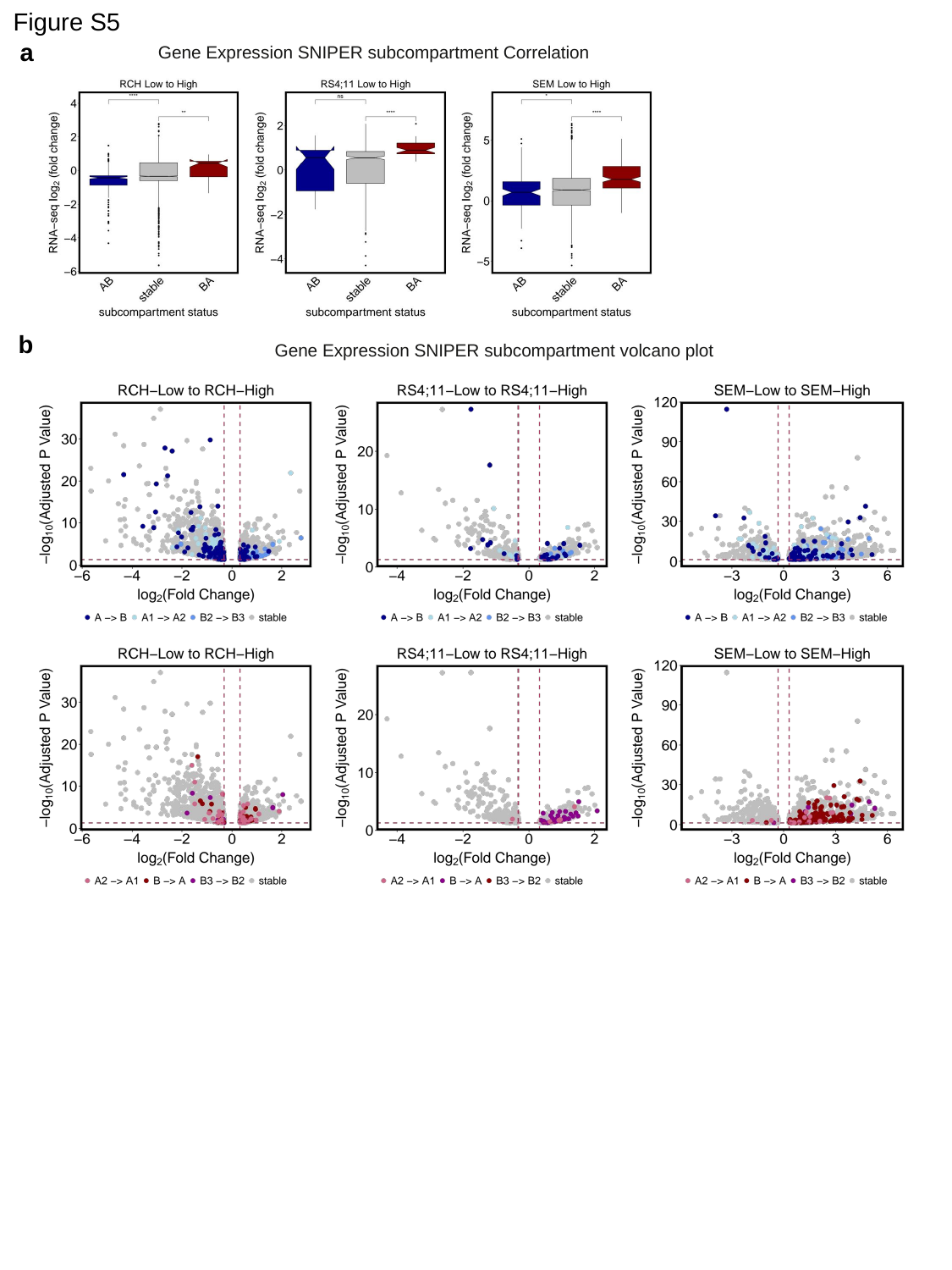

Figure S5
a
Gene Expression SNIPER subcompartment Correlation
b
Gene Expression SNIPER subcompartment volcano plot

## Slide 6
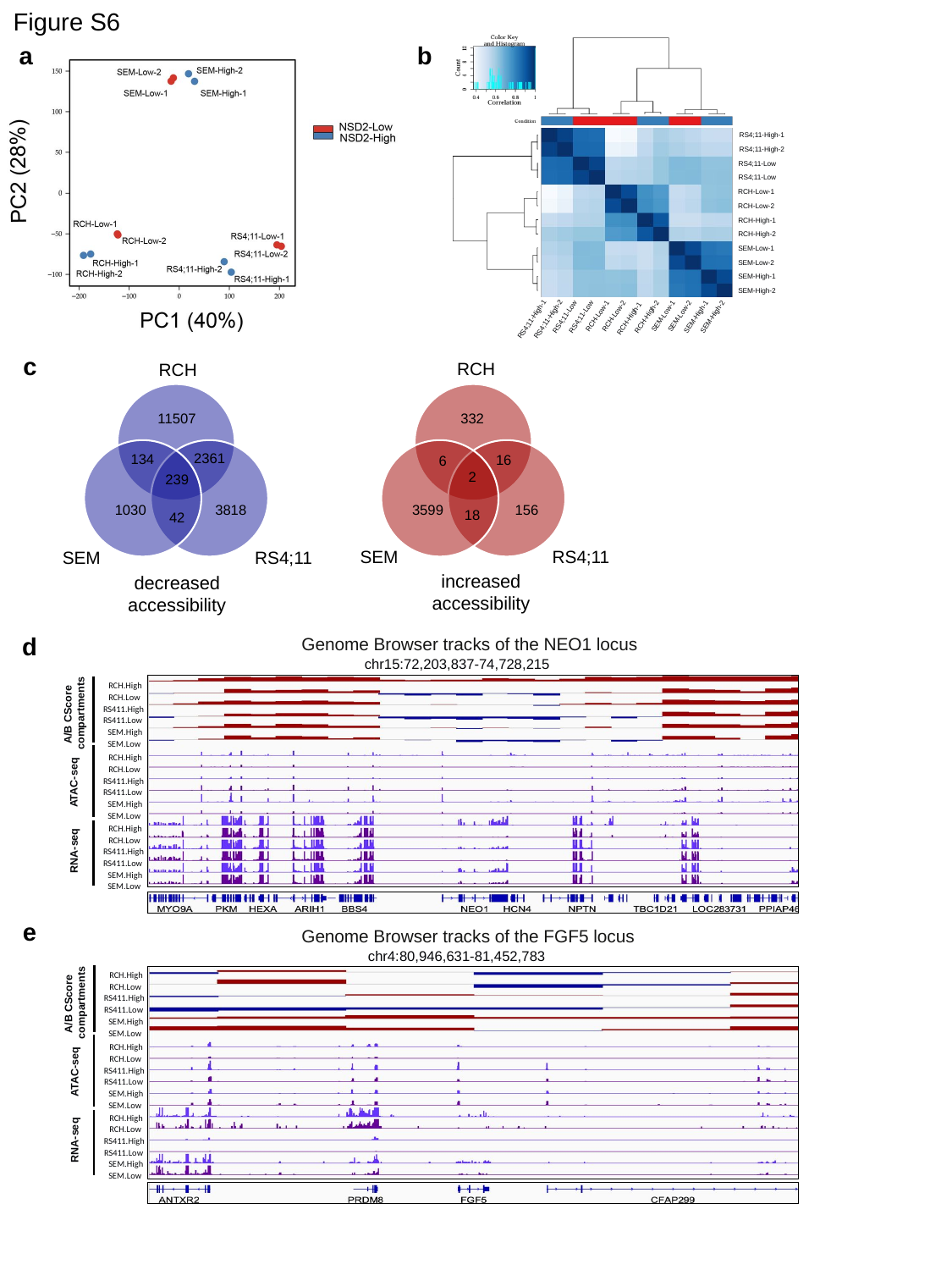

Figure S6
a
b
RS4;11-High-1
RS4;11-High-2
RS4;11-Low
RS4;11-Low
RCH-Low-1
RCH-Low-2
RCH-High-1
RCH-High-2
SEM-Low-1
SEM-Low-2
SEM-High-1
SEM-High-2
RCH-Low-1
RCH-Low-2
SEM-Low-1
SEM-Low-2
RCH-High-1
RCH-High-2
SEM-High-1
SEM-High-2
RS4;11-Low
RS4;11-Low
RS4;11-High-1
RS4;11-High-2
c
RCH
RCH
332
16
6
2
3599
156
18
11507
2361
134
239
1030
3818
42
SEM
RS4;11
SEM
RS4;11
increased
accessibility
decreased
accessibility
d
Genome Browser tracks of the NEO1 locus
RCH.High
RCH.Low
RS411.High
RS411.Low
SEM.High
SEM.Low
RCH.High
RCH.Low
RS411.High
RS411.Low
SEM.High
SEM.Low
RCH.High
RCH.Low
RS411.High
RS411.Low
SEM.High
SEM.Low
A/B CScore
compartments
ATAC-seq
RNA-seq
chr15:72,203,837-74,728,215
e
Genome Browser tracks of the FGF5 locus
RCH.High
RCH.Low
RS411.High
RS411.Low
SEM.High
SEM.Low
RCH.High
RCH.Low
RS411.High
RS411.Low
SEM.High
SEM.Low
RCH.High
RCH.Low
RS411.High
RS411.Low
SEM.High
SEM.Low
A/B CScore
compartments
ATAC-seq
RNA-seq
chr4:80,946,631-81,452,783

## Slide 7
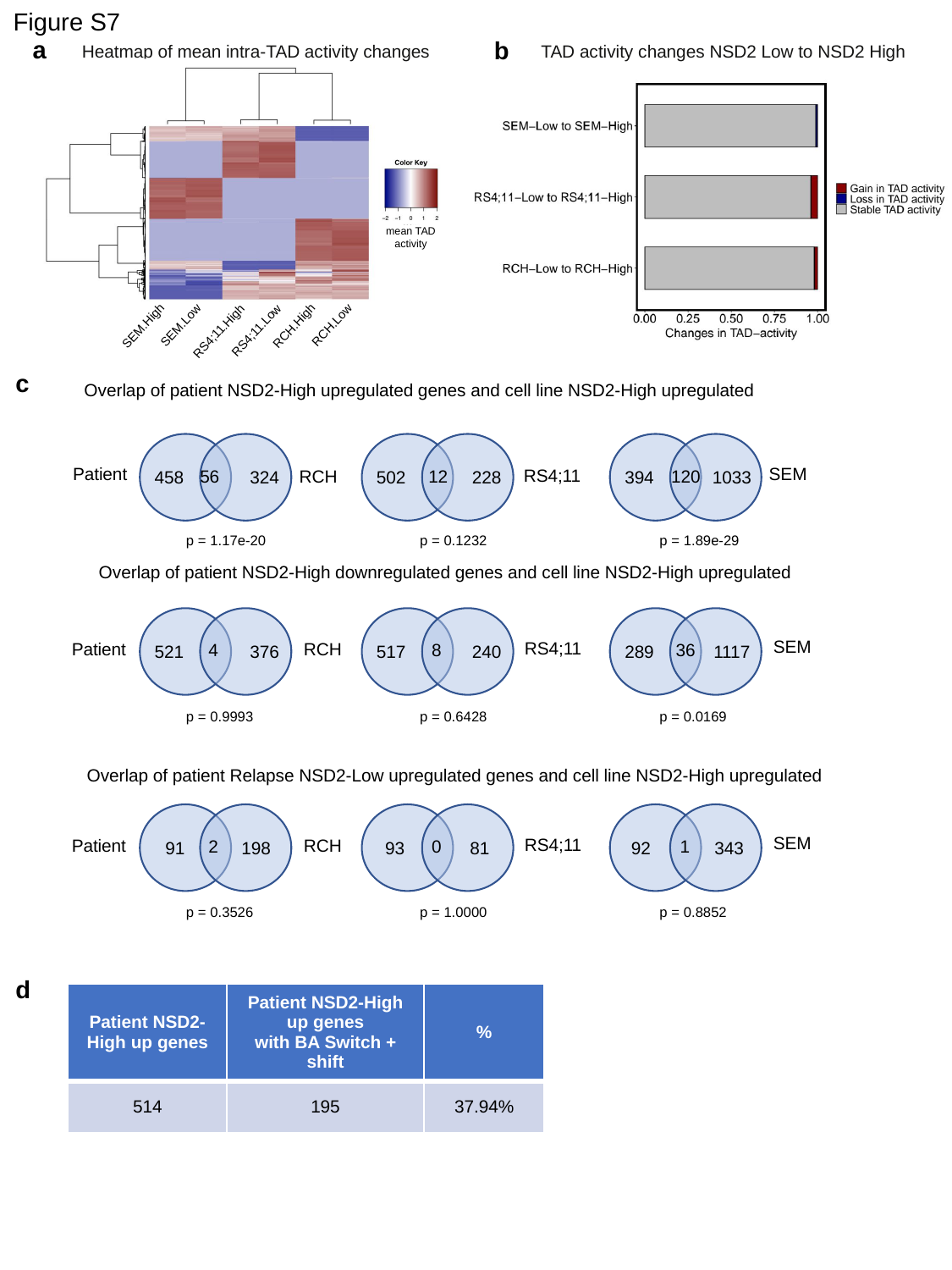

Figure S7
a
b
Heatmap of mean intra-TAD activity changes
TAD activity changes NSD2 Low to NSD2 High
mean TAD
activity
RCH.Low
SEM.Low
RCH.High
SEM.High
RS4;11.Low
RS4;11.High
c
Overlap of patient NSD2-High upregulated genes and cell line NSD2-High upregulated
56
458
324
12
502
228
120
394
1033
Patient
SEM
RS4;11
RCH
p = 1.17e-20
p = 0.1232
p = 1.89e-29
Overlap of patient NSD2-High downregulated genes and cell line NSD2-High upregulated
4
521
376
8
517
240
36
289
1117
SEM
RS4;11
Patient
RCH
p = 0.9993
p = 0.6428
p = 0.0169
Overlap of patient Relapse NSD2-Low upregulated genes and cell line NSD2-High upregulated
2
91
198
0
93
81
1
92
343
SEM
RS4;11
Patient
RCH
p = 0.3526
p = 1.0000
p = 0.8852
d
| Patient NSD2-High up genes | Patient NSD2-High up genes with BA Switch + shift | % |
| --- | --- | --- |
| 514 | 195 | 37.94% |

## Slide 8
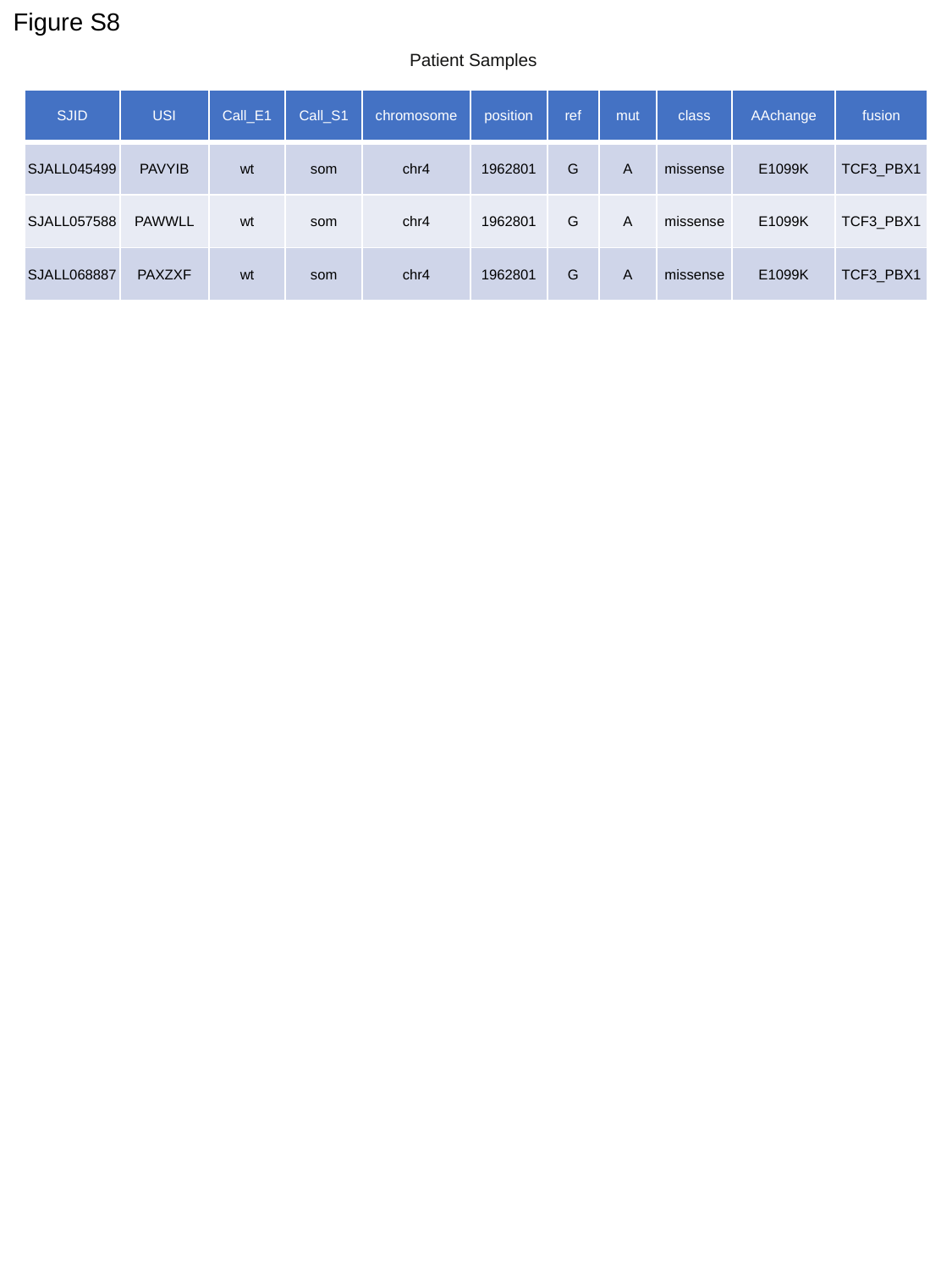

Figure S8
Patient Samples
| SJID | USI | Call\_E1 | Call\_S1 | chromosome | position | ref | mut | class | AAchange | fusion |
| --- | --- | --- | --- | --- | --- | --- | --- | --- | --- | --- |
| SJALL045499 | PAVYIB | wt | som | chr4 | 1962801 | G | A | missense | E1099K | TCF3\_PBX1 |
| SJALL057588 | PAWWLL | wt | som | chr4 | 1962801 | G | A | missense | E1099K | TCF3\_PBX1 |
| SJALL068887 | PAXZXF | wt | som | chr4 | 1962801 | G | A | missense | E1099K | TCF3\_PBX1 |

## Slide 9
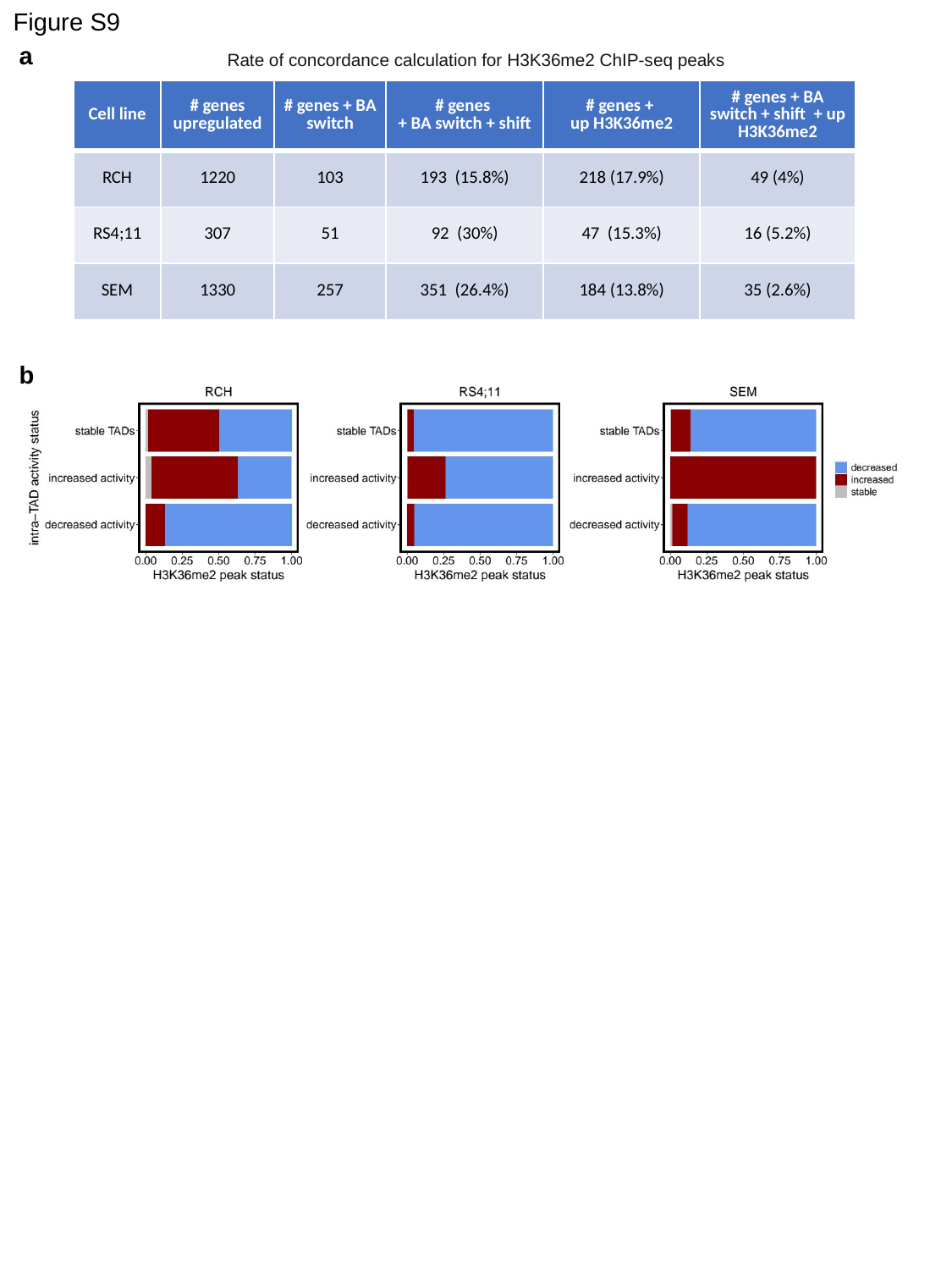

Figure S9
a
Rate of concordance calculation for H3K36me2 ChIP-seq peaks
| Cell line | # genes upregulated | # genes + BA switch | # genes + BA switch + shift | # genes + up H3K36me2 | # genes + BA switch + shift  + up H3K36me2 |
| --- | --- | --- | --- | --- | --- |
| RCH | 1220 | 103 | 193  (15.8%) | 218 (17.9%) | 49 (4%) |
| RS4;11 | 307 | 51 | 92  (30%) | 47  (15.3%) | 16 (5.2%) |
| SEM | 1330 | 257 | 351  (26.4%) | 184 (13.8%) | 35 (2.6%) |
b
