# Supplementary material for: NSD2 E1099K drives relapse in pediatric acute lymphoblastic leukemia by disrupting 3D chromatin organization
Source: Genome Biol. 2023 Apr 4;24:64. doi: 10.1186/s13059-023-02905-0 (PMC10071675; doi:10.1186/s13059-023-02905-0)
Supplement: Supplementary file 14 — Additional file 14. Review history. [file 13059_2023_2905_MOESM14_ESM.docx]

Review History

**First round of review**

**Reviewer 1**

**Were you able to assess all statistics in the manuscript, including the appropriateness of statistical tests used?**

Yes, and I have assessed the statistics in my report.

**Comments to author:**

This is an interesting study, the authors performed Hi-C on three B-ALL cell lines heterozygous for NSD2 EK and assessed changes upon knockdown. NSD2 knockdown resulted in widespread remodeling of the 3D genome, most dramatically in terms of compartment changes with a strong bias towards B compartment shifts. Systematic integration of the Hi-C data with previously published ATAC-seq, RNA-seq and ChIP-seq data showed a shrinkage in H3K36me2 and an expansion in H3K27me3 marks within B compartments as well as decreased gene expression and chromatin accessibility. The paper is generally well written and structured. However some issues still need to be addressed

1. NSD2 knockdown-related compartment switches alter gene expression in a cell-context manner, the alterations of gene expression is due to NSD2 knockdown itself or due to NSD2 knockdown-related compartment switches

2. In line 108, the authors identified NEO1 gene locus, which has been implicated in cell adhesion, the author has identified 226 shared switches in total. Were there other important gene locus identified. Why only NEO1 gene locus, the author should explain .

3. In line 216 the author acquired expression data from three matched diagnosis (NSD2 Low) and relapse (NSD2 High) patient pairs, authors may include more matched cases such as the cases with NSD2 high at diagnosis, but relapsed with NSD2 low, or the cases with NSD2 low at diagnosis,even relapsed with NSD2 Low, to check what is going on for the compartment switch or shift In the abstract , the author mentiond "this core program is an attractive target for therapeutic intervention." , so to verify the novel mechanism by which NSD2 EK drives clonal evolution is very important.

4. In line 134, how to ge t the data of 26.5% compartment changes.

minor

1. In line192 Meis1 → MEIS1

2. RS4;11/RS411 should be consistent across manuscripts.

**Reviewer 2**

**Were you able to assess all statistics in the manuscript, including the appropriateness of statistical tests used?**

Yes, and I have assessed the statistics in my report.

**Comments to author:**

This study leverage on the previous result from the author's group on the role of NSD in affecting the histone state, chromatin accessibility, and gene expression to further correlate these changes at the 3D genome scale (Pierro et al., Mol Cancer Res (2020) 18 (8): 1153-1165). The authors utilized Hi-C analysis in 3 ALL cell lines with heterozygous NSD mutation and compare the 3D genome organization changes upon knocking down the NSD gene. The authors integrated the Hi-C analysis with the previous ATAC-seq, RNA-seq and ChIP-seq data and found that there exist significant context-dependent alterations at the 3D genome level. Nevertheless, they still discovered shrinkage in H3K36me2 and an expansion in H3K27me3 marks within B compartments as well as decreased gene expression and chromatin accessibility. They thus concluded that NSD2 EK plays a prominent role in chromatin decompaction through enrichment of H3K36me2 epigenetic marks.

General comments to the study:

First, the current study is a logical extension from the author's previous work with more in-depth integrated analysis. However, this current study fell short in several aspects that might have compromised the novelty and originality of the claims.

Given the context-dependent findings, the authors claimed that the data provided a novel mechanism by which NSD2 EK drives clonal evolution. However, although the data from the 3 cell lines did show significant cell line to cell line variation, the current analysis did not elucidate sufficiently how the clonal evolution among different clones might have occurred. Therefore, the authors should tone down this claim unless there would be additional analysis on the evolution trajectory.

Following on this point of having significant cell line to cell line variations of the Hi-C data, one would start to ponder what the biological and functional significance of the selected loci shown in this study (eg. FGF5) might have in NSD mutated ALL. If the most significant finding of this study would be the shrinkage in H3K36me2 and an expansion in H3K27me3 marks within B compartments, the authors should probably dedicate more efforts on the in-depth analysis of these genomic regions to have a broader picture of the true implication.

Specific comments to each figure:

Additional file 1a: It is quite clear that the usable reads were only ~30% at best for most of the libraries with the SEM-high cell line having the lowest read counts (less than 50 million). This suggests that the quality of these Hi-C libraries was not at the most optimal level. This is of concern particularly for the SEM libraries, which should be pooled or merged with another duplicate to achieve higher read counts to at least achieve similar quality with the other 2 sets of cell lines. This could have an impact on the limit of binning size of choice. For a library with less than 50 million usable read counts, the current binning size of 40kb might not be ideal but a higher range of bin at 100kb range would be more appropriate. Therefore, for the data presented in this study, the interpretation from the SEM Hi-C results should be treated with caution.

Fig. 1d: The Y-axis denoted NSD low vs high, while the text read "We observed ~7.49% compartment switching between NSD2 High and NSD2 Low across the three cell lines (Fig. 1d).". Since there are 2 dimensions of comparison here (high vs low NSD expression and AB compartment switch), it would be clearer if the text and the figure could follow the same direction of thinking logic. A better way to show is to flip the Y-axis to NSD high to low and adjust the bar plot accordingly. Also, it is unclear how "~7.49%" was derived from this figure.

Fig. 1e: "Overall, a greater number of compartment switches occurred from A to B upon knockdown with 3,047 regions switching from A to B and 1,132 regions switching from B to A. Of the switching compartments, we identified 226 shared switches across the three cell lines from A to B and 64 from B to A (Fig. 1e)." According to the text here, the authors' definition of compartment switching direction could be from the NSD high to NSD low. Therefore, this is contradicting to the labeling of Fig. 1d.

Fig. 1f: It is not clear what the value of the color key was representing for. Z-score? The corresponding text read "30.39% (926/3047) of A to B switches and 42.93% (486/1132) B to A switches were shared by at least 2 cell lines (Fig. 1f)." which is a statement very hard to decipher from the heatmap shown here.

Fig. 1g: The figure only indicated the PRDM8 locus without labeling of the FGF5 locus.

Figure 2: The figure legends of Fig. 2b and 2c were swapped.

Fig. 2c: It seems like the AB/BA switch direction was referring to from the NSD high to NSD low following knocking NSD down but the direction of change from the RNA-seq was from NSD low to NSD high. The authors should give a clear definition of the AB/BA they are referring to throughout the manuscript and stick to it.

Fig. 2d: There were 2 "stable" being used here. To avoid confusion, the "stable" should be reserved for the compartment change whereas for the gene expression change, another term such as "no change" could be used.

Figure 3 in general & Fig. 3a: The author introduced another concept of compartment "shift" here by referring to "A to more A, B to more B, A to less A, and B to more B". It should be explained more clearly how the "more A/B, less A/B" was derived. It was not stated in either the result text or the method. Did they author utilized the PC1 values of the compartment calling? How was the cut-off determined as "having shift" versus "not having shift"? What did the values of the scatter plot represent at both the x- and y-axis? If the values were the PC1 values from the PCA plot, it is not appropriate to utilize it as such since these values were arbitrary. A better way to refine the compartment analysis is to call sub-compartments to see whether there would be switches at the sub-compartment levels within those "stable" compartments. The rest of the analysis in Figure 3 should utilize the change at the sub-compartment level instead of the "shift" concept. Having said that, the low read counts of some libraries should also be reminded here that any analysis involving finer resolution might be compromised for the low depth coverage.

Fig. 3c: To avoid confusion, the "stable" should be reserved for the compartment change whereas for the gene expression change, another term such as "no change" could be used.

Additional file 3: The author used "explainability" in the table. "Rate of Concordance" is a better term.

Fig. 4a: It is apparent that the chromatin state of SEM might be different from the other 2 cell lines which was shown in the authors' previous publication (Figure 6 Mol Cancer Res (2020) 18 (8): 1153-1165).

Fig. 4d: To avoid confusion, the "stable" should be reserved for the compartment change whereas for the gene expression change, another term such as "no change" could be used.

Fig. 4e: The motif analysis restricted to the concordant genomic loci among the ATAC-seq/compartment switch showed enrichment of the EZH2, SUZ12 were on the top of the AB compartment switch in addition to some self-renewal TFs. In addition to the genome-wide analysis on the differentially expressed genes shown in the present study, the authors should try to zoom specifically into these binding sites to run the concordant analysis of compartment switch again. Also, please incorporate a list of genomic regions with concordance changes that was used for this motif enrichment analysis.

Additional file 4d: There is no color legend, and it was not mentioned in the text for any relevance.

Figure 5 in general: The authors entered the next hierarchy of TAD here and used the concept of "intra-TAD activity" for the analysis. Even though a reference (#17) was cited here to infer "intra-TAD activity", the authors should still clarify whether this was the average of the normalized interaction scores for all interactions within the particular TAD delivered by the Hic-bench pipeline (#19) with clear elaboration of the method in the supplementary file. In addition, the analysis of the TAD could also incorporate the aspects of TAD boundaries and

Fig. 5a: It is not clear what the value of the color key was representing for.

Fig. 5b: It is apparent that the same cell line background shared similar intra-TAD activity. Can the authors use volcano plots to show the differential intra-TAD activity for each cell line pair and try to label some of the top regions? The current bar plot is confusing in terms of the directionality whether the gain/loss of intra-TAD activity was referring to NSD low versus NSD high or the other way around. Also, please explain how the "gain" and "loss" of intra-TAD activity was determined.

Fig. 5e: This is probably the most interesting data point so far for this study. There were more BA compartment switch under those increased intra-TAD activity and more AB compartment switch under those decreased intra-TAD activity. What were these genomic regions in each cell line pair? Were they enriched in any pathways or motifs? Even though these regions might not be shared among these 3 cell lines, it would still be worthwhile reporting them given the fact that the authors would also like to stress the point of "context-dependency" and "clonal heterogeneity". The authors should dive into these data more. The current data seemed to be scraping over the surface. In addition, did the compartment here include those "shift"?

Fig. 5f: There should be a genomic location plot beneath these contact matrices to show where intra-TAD interactions were the authors referring to.

Figure 7a: The top panels of each sub-figure were lacking the indication of cell line name.

Figure 7c: The top panels of each sub-figure were lacking the indication of cell line name and the comparison directionality.

Figure 7d: This is another interesting piece of data as well. The loss of H3K36me2 and gain of H3K27me3 have been reported by the author's group previously and here they showed the correlation with the compartment switch. The analysis only stopped at the % of change of the histone marks in relation to the compartment switch. There was little information advanced regarding where these genomic regions were located and what pathways/functions of these regions were referring to. In addition, how about the histone states in relation to the intra-TAD activity and TAD boundary changes? The top panels of each sub-figure were lacking the indication of cell line name and the comparison directionality. The authors only showed the data from one cell line. It would be worthwhile to look into the other 2 as well.

Additional file 7: The author used "explainability" in the table. "Rate of Concordance" is a better term.

**Authors Response**

**Point-by-point responses to the reviewers’ comments:**

Reviewer #1

This is an interesting study, the authors performed Hi-C on three B-ALL cell lines heterozygous for NSD2 EK and assessed changes upon knockdown. NSD2 knockdown resulted in widespread remodeling of the 3D genome, most dramatically in terms of compartment changes with a strong bias towards B compartment shifts. Systematic integration of the Hi-C data with previously published ATAC-seq, RNA-seq and ChIP-seq data showed a shrinkage in H3K36me2 and an expansion in H3K27me3 marks within B compartments as well as decreased gene expression and chromatin accessibility. The paper is generally well written and structured. However some issues still need to be addressed.

*Reply: We would first like to thank the reviewer for finding our study interesting. We have extensively amended this study to best address the comments from both reviewer 1 and reviewer 2. Firstly, we changed the order of each comparison to “NSD2 Low to NSD2 High” which is equivalent to “NSD2 High versus NSD2 Low”. Therefore now, we discuss how NSD2 low to NSD2 high, leads to a switch from compartment B to A (i.e. decompaction), which we find more intuitive. We generated new Hi-C data for the SEM Low and SEM High cell lines so that we achieved higher usable read counts that were similar to that of the RCH and RS4;11 cell lines (~100 million reads). Now all of our Hi-C libraries are at the most optimal level and of similar quality. Additionally, we added a subcompartments analysis to further investigate whether there were shifts at the subcompartment levels. Lastly, we generated new ChIP-seq data for the RCH and SEM cell lines since we originally only had ChIP-seq data for the RS4;11 cell line. This allowed us to assess the relationship between compartments and the H3K36me2 epigenetic mark in all three cell lines.*

*1. NSD2 knockdown-related compartment switches alter gene expression in a cell-context manner, the alterations of gene expression is due to NSD2 knockdown itself or due to NSD2 knockdown-related compartment switches.*

*From previous work, we know that NSD2 regulates H3K36me2, therefore, we can reasonably argue that the H3K36me2-related gene expression changes are due to NSD2. Under this assumption, we calculated the percentage of gene changes that can be attributed to NSD2 knockdown itself (through increased H3K36me2) or due to the NSD2 knockdown-related compartment switches (decompaction). Approximately 16-30% of upregulated genes were found within decompacted loci, while ~14-18% presented with increased H3K36me2.*

*Cell line Upregulated genes Upregulated genes in decompacted loci Upregulated genes in loci with increased H3K36me3*

*RCH 1220 193 (15.8%) 218 (17.9%)*

*RS4;11 307 92 (30%) 47 (15.3%)*

*SEM 1330 351 (26.4%) 184 (13.8%)*

2. In line 108, the authors identified NEO1 gene locus, which has been implicated in cell adhesion, the author has identified 226 shared switches in total. Were there other important gene locus identified. Why only NEO1 gene locus, the author should explain.

*Reply: This value was updated with the new Hi-C data from the SEM High and SEM Low cell lines to 219 shared switches by the three cell lines from B to A. We address this comment with the addition of two supplemental files consisting of the shared switches and the associated genes impacted by these switches in all of the cell lines, one for increased expression with B to A compartment switch and one with decreased expression with A to B compartment switch. We chose to show NEO1 because it was shared by all three cell lines and identified in the patient samples at relapse and is associated with cell adhesion which NSD2 has been demonstrated to affect. We now make this more clear in the text and also point out a few other notable genes, including EPS8 which has been associated with poor prognosis in ALL and is known to regulate proliferation and apoptosis (Huang et al., Oncology Reports 2017). FAM92A (also known as BARMR1) which has been negatively correlated with prognosis in AML and shown to promote proliferation and colony formation, and IRS1 which activates PI3K/AKT/mTOR, B-catenin, and MAPK and has been a target for inhibiting MAPK in ALL.*

3. In line 216 the author acquired expression data from three matched diagnosis (NSD2 Low) and relapse (NSD2 High) patient pairs, authors may include more matched cases such as the cases with NSD2 high at diagnosis, but relapsed with NSD2 low, or the cases with NSD2 low at diagnosis, even relapsed with NSD2 Low, to check what is going on for the compartment switch or shift

*Reply: This is a great suggestion. We note here that the NSD2 E1099K mutation is commonly seen in B-ALL patients as a relapse-specific (or enriched) mutation, and therefore this mutation has not been observed at diagnosis and then lost at relapse. The patient samples that we have consist of either NSD2 Low at diagnosis and NSD2 High at relapse (included in the initial version of our manuscript) or NSD2 Low at both diagnosis and relapse (proposed new analysis). To address the reviewer’s comment, we identified 9 matched samples of NSD2 Low at diagnosis and NSD2 Low at relapse with RNA-seq data. We performed differential expression analysis and identified 93 upregulated genes at relapse, and then compared this list of genes to the genes that were upregulated within decompacted loci in each of the cell lines (NSD2 Low to NSD2 High). As expected, and in contrast to what we found in the comparison with the NSD2-High/NSD2-High patient cohort, we found that the overlap was not significant, as shown below, supporting the specific role of NSD2 in mediating these changes.*

4. In the abstract , the author mentioned "this core program is an attractive target for therapeutic intervention." , so to verify the novel mechanism by which NSD2 EK drives clonal evolution is very important.

*Reply: We appreciate the reviewer’s comment and have added some more discussion about this. Based on the clinical importance of NSD2 in leukemias, there have been efforts to find targeted treatments, however they have been unsuccessful. We believe our previous work, along with the work herein, demonstrates the complexity of dysregulated epigenetic modifiers as their impacts are context dependent. Importantly, we have now discovered that epigenetic plasticity through the decompaction of compartments by NSD2 converges on specific downstream pathways, sometimes impacting the same gene across cell lines and patients (NEO1) or various genes within the same signaling cascade (FGF5 and GNAQ). While we have not focused on the overall biological mechanisms of these specific candidates, which would need to be validated in cell lines is beyond the score of this work, highlighting some of these genes as potential candidates has clear clinical relevance.*

5. In line 134, how to get the data of 26.5% compartment changes.

*Reply: Recalculation of this value (after repeating the Hi-C for the SEM cell lines) resulted in updating this value to 26.06%. Additionally, we now carefully describe the method that was used to calculate this value in the methods section of the manuscript:*

*“For the compartment switch analysis, percent switches in compartments was calculated by quantifying the sum of the total number of bins that switched from A to B and B to A divided by the total number of compartment bins (switching bins + stable bins). For compartment switch and shift analysis, bins were classified as A to B or B to A switches if there was a switch in the compartment score sign. Bins were classified as B to more B, B to less B, A to more A, or A to less A if there wasn’t a switch in the compartment score sign but the delta exceeded a cutoff of delta > 0.2. Additionally, percent switches in compartments was calculated by quantifying the sum of the total number of bins that switched from B to more B, B to less B, A to more A, A to less A, A to B, and B to A divided by the total number of compartment bins (switching bins + shifting bins + stable bins).”*

Minor

1. In line192 Meis1 → MEIS1.

2. RS4;11/RS411 should be consistent across manuscripts.

*Reply: We carefully reviewed every figure and supplemental figure to make corrections to these two minor points so that Meis1 is now MEIS1 and RS411 is now RS4;11 consistently throughout the manuscript, figures, and additional figures.*

Reviewer #2

This study leverage on the previous result from the author's group on the role of NSD in affecting the histone state, chromatin accessibility, and gene expression to further correlate these changes at the 3D genome scale (Pierro et al., Mol Cancer Res (2020) 18 (8): 1153-1165). The authors utilized Hi-C analysis in 3 ALL cell lines with heterozygous NSD mutation and compare the 3D genome organization changes upon knocking down the NSD gene. The authors integrated the Hi-C analysis with the previous ATAC-seq, RNA-seq and ChIP-seq data and found that there exist significant context-dependent alterations at the 3D genome level. Nevertheless, they still discovered shrinkage in H3K36me2 and an expansion in H3K27me3 marks within B compartments as well as decreased gene expression and chromatin accessibility. They thus concluded that NSD2 EK plays a prominent role in chromatin decompaction through enrichment of H3K36me2 epigenetic marks.

General comments to the study:

First, the current study is a logical extension from the author's previous work with more in-depth integrated analysis. However, this current study fell short in several aspects that might have compromised the novelty and originality of the claims. Given the context-dependent findings, the authors claimed that the data provided a novel mechanism by which NSD2 EK drives clonal evolution. However, although the data from the 3 cell lines did show significant cell line to cell line variation, the current analysis did not elucidate sufficiently how the clonal evolution among different clones might have occurred. Therefore, the authors should tone down this claim unless there would be additional analysis on the evolution trajectory. Following on this point of having significant cell line to cell line variations of the Hi-C data, one would start to ponder what the biological and functional significance of the selected loci shown in this study (eg. FGF5) might have in NSD mutated ALL. If the most significant finding of this study would be the shrinkage in H3K36me2 and an expansion in H3K27me3 marks within B compartments, the authors should probably dedicate more efforts on the in-depth analysis of these genomic regions to have a broader picture of the true implications.

*Reply: We would first like to thank the reviewer for making such meaningful suggestions. We have extensively amended this study to best address the comments from both reviewer 1 and reviewer 2. Firstly, we changed the order of each comparison to “NSD2 Low to NSD2 High” which is equivalent to “NSD2 High versus NSD2 Low”. Therefore now, we discuss how NSD2 low to NSD2 high, leads to a switch from compartment B to A (i.e. decompaction), which we find much more intuitive. We generated new Hi-C data for the SEM Low and SEM High cell lines so that we achieved higher usable read counts that were similar to that of the RCH and RS4;11 cell lines (~100 million reads). Now all of our Hi-C libraries are at the most optimal level and of similar quality. Additionally, we added a complimentary subcompartments analysis to further investigate whether there were shifts at the subcompartment levels. We agree with the reviewer that the goal of this paper was not to fully elucidate how clonal evolution among different clones might have occurred so we now tone down these claims. Lastly, we generated new ChIP-seq data for the RCH and SEM cell lines since we originally only had ChIP-seq data for the RS4;11 cell line. This allowed us to assess the relationship between A/B compartments and the H3K36me2 epigenetic mark in all three cell lines as well as perform a more in-depth analysis of these genomic regions to have a broader picture of their true implications.*

Specific comments to each figure:

Additional file 1a: It is quite clear that the usable reads were only ~30% at best for most of the libraries with the SEM-high cell line having the lowest read counts (less than 50 million). This suggests that the quality of these Hi-C libraries was not at the most optimal level. This is of concern particularly for the SEM libraries, which should be pooled or merged with another duplicate to achieve higher read counts to at least achieve similar quality with the other 2 sets of cell lines. This could have an impact on the limit of binning size of choice. For a library with less than 50 million usable read counts, the current binning size of 40kb might not be ideal but a higher range of bin at 100kb range would be more appropriate. Therefore, for the data presented in this study, the interpretation from the SEM Hi-C results should be treated with caution.

*Reply: To address this comment, we generated new Hi-C data for the SEM Low and SEM High cell lines so that the usable reads were similar to that of the RCH and RS4;11 cell lines (~100 million reads). The new alignment rate can be seen in Additional File 1a, shown below. Overall the new data strengthened our original conclusion and did not result in any major changes to our overall findings.*

Fig. 1d: The Y-axis denoted NSD low vs high, while the text read "We observed ~7.49% compartment switching between NSD2 High and NSD2 Low across the three cell lines (Fig. 1d).". Since there are 2 dimensions of comparison here (high vs low NSD expression and AB compartment switch), it would be clearer if the text and the figure could follow the same direction of thinking logic. A better way to show is to flip the Y-axis to NSD high to low and adjust the bar plot accordingly. Also, it is unclear how "~7.49%" was derived from this figure.

*Reply: We thank the reviewer for this very useful comment, which we believe will improve the clarity of our presentation. We addressed this comment by changing each comparison to “NSD2 Low to NSD2 High” which is equivalent to “NSD2 High versus NSD2 Low”. Therefore now, we discuss how NSD2 low to NSD2 high, leads to a switch from compartment B to A (i.e. decompaction), which we find much more intuitive. We further clarified the comparison in the manuscript text as follows:*

*“We performed Hi-C and systematically integrated it with previously published ATAC-seq, RNA-seq, and ChIP-seq data from three B-ALL cell lines heterozygous for NSD2 EK (RS4;11, RCH-ACV, SEM) either expressing a NSD2 targeting shRNA or a non-targeting shRNA henceforth referred to as NSD2 Low and High cell lines [5] (Fig 1a). In this study, we assessed changes in these three cell lines from NSD2 Low (control) to NSD2 High (mutant) as NSD2 EK is a relapse-enriched mutation in patients and results in the overactive form of NSD2. ”*

*Additionally, we explained how the percent change in the A/B compartments was derived in the methods section of the manuscript:*

*“For the compartment switch analysis, percent switches in compartments was calculated by quantifying the sum of the total number of bins that switched from A to B and B to A divided by the total number of compartment bins (switching bins + stable bins). For compartment switch and shift analysis, bins were classified as A to B or B to A switches if there was a switch in the compartment Cscore sign. Bins were classified as B to more B, B to less B, A to more A, or A to less A if there wasn’t a switch in the compartment Cscore sign but the delta exceeded a cutoff of delta > 0.2. Additionally, percent switches in compartments was calculated by quantifying the sum of the total number of bins that switched from B to more B, B to less B, A to more A, A to less A, A to B, and B to A divided by the total number of compartment bins (switching bins + shifting bins + stable bins).”*

Fig. 1e: "Overall, a greater number of compartment switches occurred from A to B upon knockdown with 3,047 regions switching from A to B and 1,132 regions switching from B to A. Of the switching compartments, we identified 226 shared switches across the three cell lines from A to B and 64 from B to A (Fig. 1e)." According to the text here, the authors' definition of compartment switching direction could be from the NSD high to NSD low. Therefore, this is contradicting to the labeling of Fig. 1d.

*Reply: We thank the reviewer for this comment. We believe that our inconsistent labeling has led to this confusion. We addressed this comment by adopting a more consistent labeling method changing each comparison to “NSD2 Low to NSD2 High'' which is equivalent to “NSD2 High versus NSD2 Low”. Therefore now, we discuss how NSD2 low to NSD2 high, leads to a switch from compartment B to A (i.e. decompaction), which we find much more intuitive. We believe that this consistent labeling method should clarify a number of comments related to the direction of comparison. We further described the comparison in the manuscript text as follows:*

*“We performed Hi-C and systematically integrated it with previously published ATAC-seq, RNA-seq, and ChIP-seq data from three B-ALL cell lines heterozygous for NSD2 EK (RS4;11, RCH-ACV, SEM) either expressing a NSD2 targeting shRNA or a non-targeting shRNA henceforth referred to as NSD2 Low and High cell lines [5] (Fig 1a). In this study, we assessed changes in these three cell lines from NSD2 Low (control) to NSD2 High (mutant) as NSD2 EK is a relapse-enriched mutation in patients and results in the overactive form of NSD2. ”*

*This text now reads as the following:*

*“Overall, a greater number of compartment switches occurred from B to A with 3,047 regions switching from B to A and 1,132 regions switching from A to B. Of the switching compartments, we identified 226 shared switches across the three cell lines from B to A and 64 from A to B (Fig. 1e).”*

*Figure 1d was also updated to reflect these changes:*

*Fig. 1f: It is not clear what the value of the color key was representing for. Z-score? The corresponding text read "30.39% (926/3047) of A to B switches and 42.93% (486/1132) B to A switches were shared by at least 2 cell lines (Fig. 1f)." which is a statement very hard to decipher from the heatmap shown here.*

*The value of the color key for the heatmap presents the raw Cscore (not a normalized z-score) associated with each genomic bin that was involved in a switch shared by at least 2 of the 3 cell lines. For example, if a genomic window switched from A to B, and this genomic window was also switching from A to B in an additional cell line, its Cscore value was included in the heatmap. We now more clearly label the color key as “Cscore'' as follows:*

*Additionally, we describe what Cscore is in the methods section as follows:*

*A/B compartments analysis was performed using Cscore tool. The Cscore tool algorithm was used to assign active (A) and inactive (B) compartments [20]. Cscore assumes that each genomic window, i, has a chance Pi to be in the A-compartment. By defining Cscore as Ci=2Pi−1, which ranges between −1 and 1, Cscore deduces a log-likelihood function where nij is the observed number of contacts, dij is the distance along the genome, H(dij) is the scaling factor accounting for the decrease of interactions at longer genomic distance, and Bi and Bj are the bias factors from Hi-C experiments. A maximum-likelihood estimation was performed for the model parameters B, C and H using an iterative algorithm:*

Fig. 1g: The figure only indicated the PRDM8 locus without labeling of the FGF5 locus.

*Reply: This has been corrected with the addition of the FGF5 label as follows:*

Figure 2: The figure legends of Fig. 2b and 2c were swapped.

*Reply: We thank the reviewer for pointing this out. We have now corrected this mistake. The figure legend for Figure 2 now reads as:*

*“Figure 2. NSD2 EK-related B to A compartment switches correlate with gene expression changes. a. PCA representation of triplicate RNA-seq data showing top 1000 genes. b. Volcano plots demonstrating differentially expressed genes (abs(L2FC) > 0.58, p-value < 0.05) per cell line from NSD2 Low to NSD2 High with top 10 labeled. c. Correlation boxplots of gene expression changes and compartment switch status (AB, BA, stable) for each cell line. d. Association barplots showing fraction of genes (increased, decreased, stable) at compartment switches (AB, BA, stable). ”*

Fig. 2c: It seems like the AB/BA switch direction was referring to from the NSD high to NSD low following knocking NSD down but the direction of change from the RNA-seq was from NSD low to NSD high. The authors should give a clear definition of the AB/BA they are referring to throughout the manuscript and stick to it.

*Reply: We believe that our inconsistent labeling has led to this confusion. We addressed this comment by adopting a more consistent labeling method changing each comparison to “NSD2 Low to NSD2 High'' which is equivalent to “NSD2 High versus NSD2 Low”. Therefore now, we discuss how NSD2 low to NSD2 high, leads to a switch from compartment B to A (i.e. decompaction), which we find much more intuitive. We believe that this consistent labeling method should clarify a number of comments related to the direction of comparison. We further described the comparison in the manuscript text as follows:*

*“We performed Hi-C and systematically integrated it with previously published ATAC-seq, RNA-seq, and ChIP-seq data from three B-ALL cell lines heterozygous for NSD2 EK (RS4;11, RCH-ACV, SEM) either expressing a NSD2 targeting shRNA or a non-targeting shRNA henceforth referred to as NSD2 Low and High cell lines [5] (Fig 1a). In this study, we assessed changes in these three cell lines from NSD2 Low (control) to NSD2 High (mutant) as NSD2 EK is a relapse-enriched mutation in patients and results in the overactive form of NSD2. ”*

Fig. 2d: There were 2 "stable" being used here. To avoid confusion, the "stable" should be reserved for the compartment change whereas for the gene expression change, another term such as "no change" could be used.

*Reply: This comment was addressed by changing “stable” gene expression to “no change” as follows in figure 2d:*

Figure 3 in general & Fig. 3a: The author introduced another concept of compartment "shift" here by referring to "A to more A, B to more B, A to less A, and B to more B". It should be explained more clearly how the "more A/B, less A/B" was derived. It was not stated in either the result text or the method. Did they author utilized the PC1 values of the compartment calling? How was the cut-off determined as "having shift" versus "not having shift"? What did the values of the scatter plot represent at both the x- and y-axis? If the values were the PC1 values from the PCA plot, it is not appropriate to utilize it as such since these values were arbitrary. A better way to refine the compartment analysis is to call sub-compartments to see whether there would be switches at the sub-compartment levels within those "stable" compartments. The rest of the analysis in Figure 3 should utilize the change at the sub-compartment level instead of the "shift" concept. Having said that, the low read counts of some libraries should also be reminded here that any analysis involving finer resolution might be compromised for the low depth coverage.

*Reply: To address the reviewer’s comment, we first provide more details on the concept of compartment shifts, and then we proceed with a subcompartment analysis as suggested.*

*We utilized the concept of compartment shifts because the compartment switch analysis was not sufficient to capture the entire picture of compartment changes, especially compaction and decompaction. This type of analysis has been used previously by our lab and others, see for example (Yusufova et al., Nature 2021). In this work, compartment “shifts” are described as “decompaction/compaction within respective compartments” as shown below:*

*In order to address this comment, we have provided a much more detailed explanation of the compartment switch/shift analysis in the methods section as follows:*

*“For compartment switch and shift analysis, bins were classified as A to B or B to A switches if there was a switch in the compartment Cscore sign. Bins were classified as B to more B, B to less B, A to more A, or A to less A if there wasn’t a switch in the compartment Cscore sign but the delta exceeded a cutoff of delta > 0.2. Additionally, percent switches in compartments was calculated by quantifying the sum of the total number of bins that switched from B to more B, B to less B, A to more A, A to less A, A to B, and B to A divided by the total number of compartment bins (switching bins + shifting bins + stable bins).”*

*Additionally, we performed a subcompartments analysis by applying SNIPER to our Hi-C datasets as a complementary analysis to the compartments switch and shift analysis (Additional File 3 and 4). SNIPER (Subcompartment iNference using Imputed Probabilistic ExpRessions), is a computational method that is based on denoising autoencoder and multilayer perceptron classifier to infer subcompartments using typical Hi-C datasets with moderate coverage. SNIPER reveals subcompartments using moderate coverage Hi-C datasets.*

*We first present the number of subcompartments classified as A1, A2, B1, B2, and B3 with SNIPER for each of our cell lines at NSD2 Low and NSD2 High (Reviewer Fig. 1a). Next, we did a head-to-head comparison of the SNIPER subcompartments analysis with our initial analysis using Cscore. For this analysis, we identified the fraction of Cscore A or B compartments that overlapped the SNIPER subcompartments (Reviewer Fig. 1b). This data revealed that the B1 subcompartment was made up of mostly Cscore compartment A suggesting a discrepancy between the two methods. Moving forward, we excluded subcompartment B1 in order to investigate the SNIPER subcompartment analysis further. We present the number of subcompartments classified as A1, A2, B2, and B3 with SNIPER for each of our cell lines at NSD2 Low and NSD2 High (Reviewer Fig. 1c).*

*We next examined changes in A/B subcompartments between NSD2 Low and NSD2 High cell lines (Reviewer Fig. 1d). We noticed that both the RS4;11 and SEM cell lines showed a greater number of B to A subcompartment switches than A to B from NSD2 Low to NSD2 High which was consistent with our Cscore analysis.. Next, we further classified subcompartment calls into shifts in addition to subcompartment switches. Activating subcompartment shifts include B3 to B1, B3 to B2, B2 to B1, and A2 to more A1. Deactivating subcompartment shifts include B1 to B3, B2 to B3, B1 to B2, and A1 to more A2. With this data, we identified the fraction of SNIPER subcompartment switches and shifts that overlapped the Cscore compartments switches and shifts (Reviewer Fig. 1e). Overall, this data showed similar trends in Cscore compartments and SNIPER subcompartments switches and shifts.*

*Lastly, to examine the relationship between alterations in SNIPER subcompartments and gene expression, subcompartment switches were categorized as A to B, B to A, or stable and then assessed for changes in gene expression. B to A subcompartment switches seemed to more significantly correlate with upregulated genes than A to B subcompartment switches did with downregulated genes as we saw with the Cscore and gene expression analysis (Reviewer Fig. 2a). To further examine the relationship between alterations in SNIPER subcompartments and gene expression, we added subcompartment shifts in addition to switches. We identified that the majority of B to A switches and shifts were made up of significantly upregulated genes (Reviewer Fig. 2b (bottom row)), whereas A to B switches and shifts did not demonstrate a clear pattern similar to our findings with Cscore switches and shifts (Reviewer Fig. 2b (top row)).*

Fig. 3c: To avoid confusion, the "stable" should be reserved for the compartment change whereas for the gene expression change, another term such as "no change" could be used.

*Reply: This comment was addressed by changing “stable” gene expression to “no change” as follows in figure 3c:*

*Additional file 3: The author used "explainability" in the table. "Rate of Concordance" is a better term.*

*We addressed this comment by referring to the calculation as “Rate of concordance” both in the manuscript tables and in the manuscript text.*

Fig. 4a: It is apparent that the chromatin state of SEM might be different from the other 2 cell lines which was shown in the authors' previous publication (Figure 6 Mol Cancer Res (2020) 18 (8): 1153-1165).

*Reply: We have also commented on this in the text and in the discussion.*

Fig. 4d: To avoid confusion, the "stable" should be reserved for the compartment change whereas for the gene expression change, another term such as "no change" could be used.

*Reply: This comment was addressed by changing “stable” gene expression to “no change” as follows in figure 4d:*

*Fig. 4e: The motif analysis restricted to the concordant genomic loci among the ATAC-seq/compartment switch showed enrichment of the EZH2, SUZ12 were on the top of the AB compartment switch in addition to some self-renewal TFs. In addition to the genome-wide analysis on the differentially expressed genes shown in the present study, the authors should try to zoom specifically into these binding sites to run the concordant analysis of compartment switch again. Also, please incorporate a list of genomic regions with concordance changes that was used for this motif enrichment analysis.*

*To identify key regulators of transcription attributed to compartment switches, we performed an enrichment analysis with LOLA using two lists of genomic loci: 1. concordant increased ATAC-seq peaks, increased gene expression, and B to A compartment switches and shifts. 2. concordant decreased ATAC-seq peaks, decreased gene expression, and A to B compartment switches and shifts (Fig. 4e). We have also addressed this comment by providing four additional files, three of which consist of these genomic loci used as input for the LOLA analysis and one of which consists of the output enrichment results from the LOLA analysis. We believe this is what the reviewer is requesting. If there is anything else, we would be happy to address it.*

Additional file 4d: There is no color legend, and it was not mentioned in the text for any relevance.

*Reply: This comment has been addressed with the removal of this extra figure that was not relevant.*

Figure 5 in general: The authors entered the next hierarchy of TAD here and used the concept of "intra-TAD activity" for the analysis. Even though a reference (#17) was cited here to infer "intra-TAD activity", the authors should still clarify whether this was the average of the normalized interaction scores for all interactions within the particular TAD delivered by the Hic-bench pipeline (#19) with clear elaboration of the method in the supplementary file.

*Reply: This comment is addressed with the clarification of this method in the intra-TAD activity methods section in the manuscript text:*

*“Intra-TAD activity was assessed using the hic-ratio algorithm developed within hic-bench. Intra-TAD activity was assessed using the “domains” and “domains-diff” steps within the hic-bench platform. The “domains” step uses the hic-ratio algorithm for TAD calling developed within hic-bench by previous Tsirigos lab member Haris Lazaris in which the average of the normalized interaction scores is calculated for all interactions taking place within a particular TAD. The “domains-diff” step assesses intra-TAD activity alterations and was developed by Tsirigos lab members Sofia Nomikou and Andreas Kloetgen. To identify TADs with differential activity between NSD2 Low and High cell lines, we used the TADs identified in the NDS2 High sample as a reference to identify common TADs. Once intra-TAD activity values are obtained from the Hi-C data for NSD2 Low and NSD2 High cell lines, a wilcoxon two-sided rank sum non-parametric test is performed to determine the p-values for each TAD. Multiple testing is used to correct these p-values by adjusting to the total number of TADs. Lastly, the log2 fold-change (log2FC) of intra-TAD activity value is calculated between the samples. Intra-TAD activity alterations were categorized as significant if abs(L2FC > 0.25) and FDR < 0.01.”*

Fig. 5a: It is not clear what the value of the color key was representing for.

*Reply: The value for the heatmap represents the mean intra-tad activity. This figure has been pushed to Additional file 7. Additionally, this has been clarified in the label of the figure and commented on in the figure legend as follows:*

*“Additional file 7. a. Heatmap representation of mean intra-TAD activity per cell line. b. Bar plot showing number of TAD activity changes per cell line (abs(L2FC) > 0.25, FDR < 0.01). c. Venn diagrams demonstrating significant overlap of patient NSD2-High upregulated genes and cell line NSD2-High upregulated genes and insignificant overlap of patient NSD2-High downregulated genes and cell line NSD2-High upregulated genes (one-tailed Fisher’s exact test). d. Table presenting percentage of patient NSD2-High upregulated genes overlapping a compartment switch/shift from any of the three cell lines. ”*

*The methods section describes the mean intra-TAD activity calculation as follows:*

*“Intra-TAD activity was assessed using the hic-ratio algorithm developed within hic-bench. Intra-TAD activity was assessed using the “domains” and “domains-diff” steps within the hic-bench platform. The “domains” step uses the hic-ratio algorithm for TAD calling developed within hic-bench by previous Tsirigos lab member Haris Lazaris in which the average of the normalized interaction scores is calculated for all interactions taking place within a particular TAD. The “domains-diff” step assesses intra-TAD activity alterations and was developed by Tsirigos lab members Sofia Nomikou and Andreas Kloetgen. To identify TADs with differential activity between NSD2 Low and High cell lines, we used the TADs identified in the NDS2 High sample as a reference to identify common TADs. Once intra-TAD activity values are obtained from the Hi-C data for NSD2 Low and NSD2 High cell lines, a Wilcoxon two-sided rank sum non-parametric test is performed to determine the p-values for each TAD. Multiple testing is used to correct these p-values by adjusting to the total number of TADs. Lastly, the log2 fold-change (log2FC) of intra-TAD activity value is calculated between the samples. Intra-TAD activity alterations were categorized as significant if abs(L2FC > 0.25) and FDR < 0.01.”*

*The heatmap legend has also been revised as follows:*

*Fig. 5b: It is apparent that the same cell line background shared similar intra-TAD activity. Can the authors use volcano plots to show the differential intra-TAD activity for each cell line pair and try to label some of the top regions? The current bar plot is confusing in terms of the directionality whether the gain/loss of intra-TAD activity was referring to NSD low versus NSD high or the other way around. Also, please explain how the "gain" and "loss" of intra-TAD activity was determined.*

*We believe that our inconsistent labeling has led to this confusion. We addressed this comment by adopting a more consistent labeling method changing each comparison to “NSD2 Low to NSD2 High'' which is equivalent to “NSD2 High versus NSD2 Low”. Therefore now, we discuss how NSD2 low to NSD2 high, leads to a switch from compartment B to A (i.e. decompaction), which we find much more intuitive. We believe that this consistent labeling method should clarify a number of comments related to the direction of comparison.*

*Additionally, the barplot has been pushed to supplementary and new volcano plots have been added to main figure 5:*

Fig. 5e: This is probably the most interesting data point so far for this study. There were more BA compartment switch under those increased intra-TAD activity and more AB compartment switch under those decreased intra-TAD activity. What were these genomic regions in each cell line pair? Were they enriched in any pathways or motifs? Even though these regions might not be shared among these 3 cell lines, it would still be worthwhile reporting them given the fact that the authors would also like to stress the point of "context-dependency" and "clonal heterogeneity". The authors should dive into these data more. The current data seemed to be scraping over the surface. In addition, did the compartment here include those "shift"?

*Reply: We thank the reviewer for finding this data interesting. Integrating intra-TAD activity data with compartment data did include both compartment shifts and switches. To make this figure more clear, we have revised the categories so that compartment switches from B to A, compartment shifts A to more A, and B to less B are designated as “decompaction” and compartment switches A to B, compartment shifts B to more B, and A to less A are designated as “compaction” as shown in the figure below:*

*This comment has also been addressed with the addition of 6 files. First, we have included two supplementary files with the genomic regions with TAD activity increases and decreases for all three cell lines from NSD2 Low to NSD2 High. Second, we have included two supplementary files, one including genomic regions with TAD activity increases along with gene expression increases within those TADs and one including genomic regions with TAD activity decreases along with gene expression decreases within those TADs for all three cell lines from NSD2 Low to NSD2 High. Lastly, we have included two supplementary files, one including genomic regions with TAD activity increases along with gene expression increases within those TADs and B to A compartment switches and shifts. The other file including genomic regions with TAD activity decreases along with gene expression decreases within those TADs and A to B compartment switches and shifts for all three cell lines from NSD2 Low to NSD2 High.*

*We did attempt pathway enrichment analysis with these genes but because there were too few genes, we did not identify significantly enriched pathways. This further demonstrates that the major event to be focussed on in the context of NSD2 is the A/B compartment changes.*

Fig. 5f: There should be a genomic location plot beneath these contact matrices to show where intra-TAD interactions were the authors referring to.

*Reply: We have added the genomic location plot below the contact matrices in Figure 5 as follows:*

Figure 7a: The top panels of each sub-figure were lacking the indication of cell line name.

*Reply: For Figure 7, we initially only had ChIP-seq data for one cell line (RS4;11). Now we have performed ChIP-seq on all three cell lines which are properly labeled in Figure 7.*

Figure 7c: The top panels of each sub-figure were lacking the indication of cell line name and the comparison directionality.

*Reply: For Figure 7, we initially only had ChIP-seq data for one cell line (RS4;11). Now we have performed ChIP-seq on all three cell lines which are properly labeled in figure 7. We also have consistently labeled all comparisons as “NSD2 Low to NSD2 High”.*

Figure 7d: This is another interesting piece of data as well. The loss of H3K36me2 and gain of H3K27me3 have been reported by the author's group previously and here they showed the correlation with the compartment switch. The analysis only stopped at the % of change of the histone marks in relation to the compartment switch. There was little information advanced regarding where these genomic regions were located and what pathways/functions of these regions were referring to. In addition, how about the histone states in relation to the intra-TAD activity and TAD boundary changes? The top panels of each sub-figure were lacking the indication of cell line name and the comparison directionality. The authors only showed the data from one cell line. It would be worthwhile to investigate the other 2 as well.

*Reply: For Figure 7, we initially only presented ChIP-seq data for one cell line (RS4;11). We addressed this comment by performing ChIP-seq on all three cell lines (including RCH and SEM) and this data is now included in main Figure 7. We updated the manuscript text to reflect this new data analysis as follows:*

*“NSD2 EK-mediated epigenetic changes correlate with 3D genome changes. We previously performed ChIP-seq on the RS4;11 NSD2 Low and NSD2 High cell lines [5]. For this work, we additionally performed ChIP-seq on the RCH and SEM NSD2 Low and NSD2 High cell lines for H3K36me2, H3K27me3, and H3K27ac epigenetic marks. As noted previously H3K36me2 distribution shifted from predominantly promoter regions to intergenic regions in all three cell lines, most dramatically in the RS4;11 cell line (Fig. 7a). Conversely, H3K27me3 peaks shifted from intergenic regions to promoter regions (Fig. 7a). Notably, H3K27ac was found to be enriched at promoter regions with little difference in distribution (Fig. 7a).*

*To examine the relationship between alterations in the distribution of H3K36me2 and A/B compartment switches, we categorized the A/B compartment switches into those that are AB, BA, or stable and assessed changes in H3K36me2. H3K36me2 peaks positively correlated with BA switches more significantly than with AB switches in each of the cell lines (Fig. 7c). We also identified that B to A compartment switches were associated with increased H3K36me2 (Fig. 7c). This data suggests that expansion of H3K36me2 as a result of NSD2 EK provides a more open chromatin landscape.*

*Additionally, to better understand the impact these changes have on downstream signaling, we performed pathway enrichment analysis for those differentially expressed genes that were associated with a compartment switch from B to A and an increase in H3K36me2 in any of the cell lines (Fig. 7d). Interestingly, Wnt signaling and cell-adhesion were identified as the two most significant pathways.*

*Lastly, we demonstrate an example of a genomic region that is characterized by a B to A compartment switch, increased H3K36me2 peaks, and increased gene expression at the NCAM1 gene locus in all three cell lines (Fig. 7e). This example clearly shows the relationship between NSD2 EK-mediated epigenetic modifications and 3D genome organization. NSD2 EK-mediated epigenetic changes correlate with 3D genome changes. We previously performed ChIP-seq on the RS4;11 NSD2 Low and NSD2 High cell lines [5]. For this work, we additionally performed ChIP-seq on the RCH and SEM NSD2 Low and NSD2 High cell lines for H3K36me2, H3K27me3, and H3K27ac epigenetic marks. As noted previously H3K36me2 distribution shifted from predominantly promoter regions to intergenic regions in all three cell lines, most dramatically in the RS4;11 cell line (Fig. 7a). Conversely, H3K27me3 peaks shifted from intergenic regions to promoter regions (Fig. 7a). Notably, H3K27ac was found to be enriched at promoter regions with little difference in distribution (Fig. 7a).*

*To examine the relationship between alterations in the distribution of H3K36me2 and A/B compartment switches, we categorized the A/B compartment switches into those that are AB, BA, or stable and assessed changes in H3K36me2. H3K36me2 peaks positively correlated with BA switches more significantly than with AB switches in each of the cell lines (Fig. 7c). We also identified that B to A compartment switches were associated with increased H3K36me2 (Fig. 7c). This data suggests that expansion of H3K36me2 as a result of NSD2 EK provides a more open chromatin landscape.*

*Additionally, to better understand the impact these changes have on downstream signaling, we performed pathway enrichment analysis for those differentially expressed genes that were associated with a compartment switch from B to A and an increase in H3K36me2 in any of the cell lines (Fig. 7d). Interestingly, Wnt signaling and cell-adhesion were identified as the two most significant pathways.*

*Lastly, we demonstrate an example of a genomic region that is characterized by a B to A compartment switch, increased H3K36me2 peaks, and increased gene expression at the NCAM1 gene locus in all three cell lines (Fig. 7e). This example clearly shows the relationship between NSD2 EK-mediated epigenetic modifications and 3D genome organization.”*

*Additionally, to advance our understanding of the impact these changes have on downstream signaling, we performed pathway enrichment analysis for those differentially expressed genes that were associated with a compartment switch from B to A and an increase in H3K36me2 in any of the cell lines and added the following subfigure to figure 7.*

*To address the final comment related to the ChIP-seq data, we integrated the H3K36me2 data with our intra-TAD activity data and included the results in the manuscript text and Additional figure 9b as follows:*

*“Next, to examine the relationship between alterations in the distribution of H3K36me2 and intra-TAD activity, we categorized the intra-TAD activity changes into those that are decreased, increased, or stable and assessed changes in H3K36me2. Although there were few intra-TAD activity changes, we observed that increased intra-TAD activity predominantly consisted of increased H3K36me2 and that decreased intra-TAD activity predominantly consisted of decreased H3K36me2 (Additional figure 9b).“*

Additional file 7: The author used "explainability" in the table. "Rate of Concordance" is a better term.

*Reply: We completely agree with the reviewer’s opinion and have addressed this comment by referring to the calculation as “Rate of concordance” both in the tables and in the manuscript text.*

**Second round of review**

**Reviewer 1**

The author had a good response to last comments, no further comments on the revised manuscprit.

**Reviewer 2**

In this revision, the authors have addressed the queries and made significant improvement of the manuscript to clarify the confusions. I have no further suggestions.
